# ReScale4DL: balancing pixel and contextual information for enhanced bioimage segmentation

**DOI:** 10.1038/s41467-026-77930-1

**Published:** 2026-09-18

**Authors:** Mariana G. Ferreira, Bruno M. Saraiva, António D. Brito, Mariana G. Pinho, Ricardo Henriques, Estibaliz Gómez-de-Mariscal

**Affiliations:** 1 AI-driven Optical Biology Laboratory, Instituto de Tecnologia Química e Biológica António Xavier, Universidade Nova de Lisboa, Oeiras, Portugal; 2 Bacterial Cell Biology Laboratory, Instituto de Tecnologia Química e Biológica António Xavier, Universidade Nova de Lisboa, Oeiras, Portugal; 3 UCL Laboratory for Molecular Cell Biology, University College London, London, UK; 4 NOVA Institute for Medical Systems Biology (NIMSB), Universidade Nova de Lisboa, Lisboa, Portugal; 5 iNOVA4Health, Programme in Translational Medicine, Lisboa, Portugal

**Keywords:** Microbiology, Cell biology, Machine learning

## Abstract

Deep learning is the state-of-the-art approach for bioimage segmentation. However, it presents a paradox regarding image resolution: counterintuitively, deep learning segmentation performance can improve with lower image resolutions. This phenomenon is particularly significant in microscopy, where high-resolution acquisitions come with substantial costs in throughput, storage requirements and potential photodamage. We systematically evaluate how image resolution impacts segmentation by training popular architectures on datasets downsampled to 6-50% of their original resolution, mimicking lower-magnification acquisitions. Compared with models trained on native-resolution images, segmentation accuracy either improves (by up to 25% of mean Intersection over Union (IoU)) or degrades minimally (< 5% of mean IoU) when using images downsampled by up to fourfold (25% of the original resolution). Downsampling proportionally increases information throughput while reducing storage requirements and inference time. These findings provide practical guidelines for creating efficient, sustainable and cost-effective bioimaging pipelines that reduce data and computing needs while optimising microscopy techniques.

## Abstract (web summary)

Deep-learning segmentation accuracy depends on image resolution, with each architecture having an optimum, usually at a lower microscopy resolution. Here, the authors present ReScale4DL, a framework for optimising imaging experiments by selecting resolutions to balance accuracy, throughput and cost.

## Introduction

Modern microscopy technologies have driven an unprecedented expansion in data volume. High-resolution techniques, particularly super-resolution microscopy^1^, generate substantially larger datasets than traditional methods when imaging equivalent sample areas. A single 3D multichannel cell image from a spinning disk confocal requires approximately 1GB of storage (considering a size of 4MB for each slice of a three-channel and 100 *z*-slices volume). In contrast, the same field acquired with a super-resolution laser scanning microscope would be approximately 60 times larger (estimated from images acquired in-house). To address the challenges in acquisition, storage, and processing posed by these volumes of data, researchers increasingly rely on deep learning approaches that automate analytical tasks while delivering reliable and reproducible results. For this, the bioimage analysis community has developed numerous user-friendly solutions^2,3^, including standalone software and platform-integrated plugins^4–11^, enabling researchers to leverage these powerful methodologies.

Yet, bioimage analysis faces unique efficiency challenges compared to other deep learning domains. The most significant bottlenecks include: limited computational resources and storage capacity, particularly problematic for time-lapse volumetric data; scarcity of large annotated datasets essential to train robust models and transfer learning^12,13^; and insufficient understanding of the relationship between image quality metrics and computational performance requirements^14,15^.

In computer vision, image quality often equates to an algorithm’s efficiency in extracting relevant information under hardware constraints. Counterintuitively, a low-resolution image containing sufficient detail to perform the computational task accurately may be optimal, as it maximises the information-to-resource ratio. For clarity, we define image resolution as the information content per pixel, separate from optical resolution considerations and directly relatable to pixel sizes, defined as the physical measurement of one pixel. For instance, while cell nuclei display heterogeneous structures at high resolutions, they are often more accurately segmented at lower resolutions, where sufficient contextual information can be captured using fewer pixels. This computational perspective contrasts with traditional microscopy practices, where image quality has been equated with higher resolution and magnification, assuming finer details provide more informative content^1^. However, while visually appealing, the wealth of information in high-resolution images can be irrelevant or even detrimental to computational analysis, as our results here demonstrate.

**Fig. 1:**
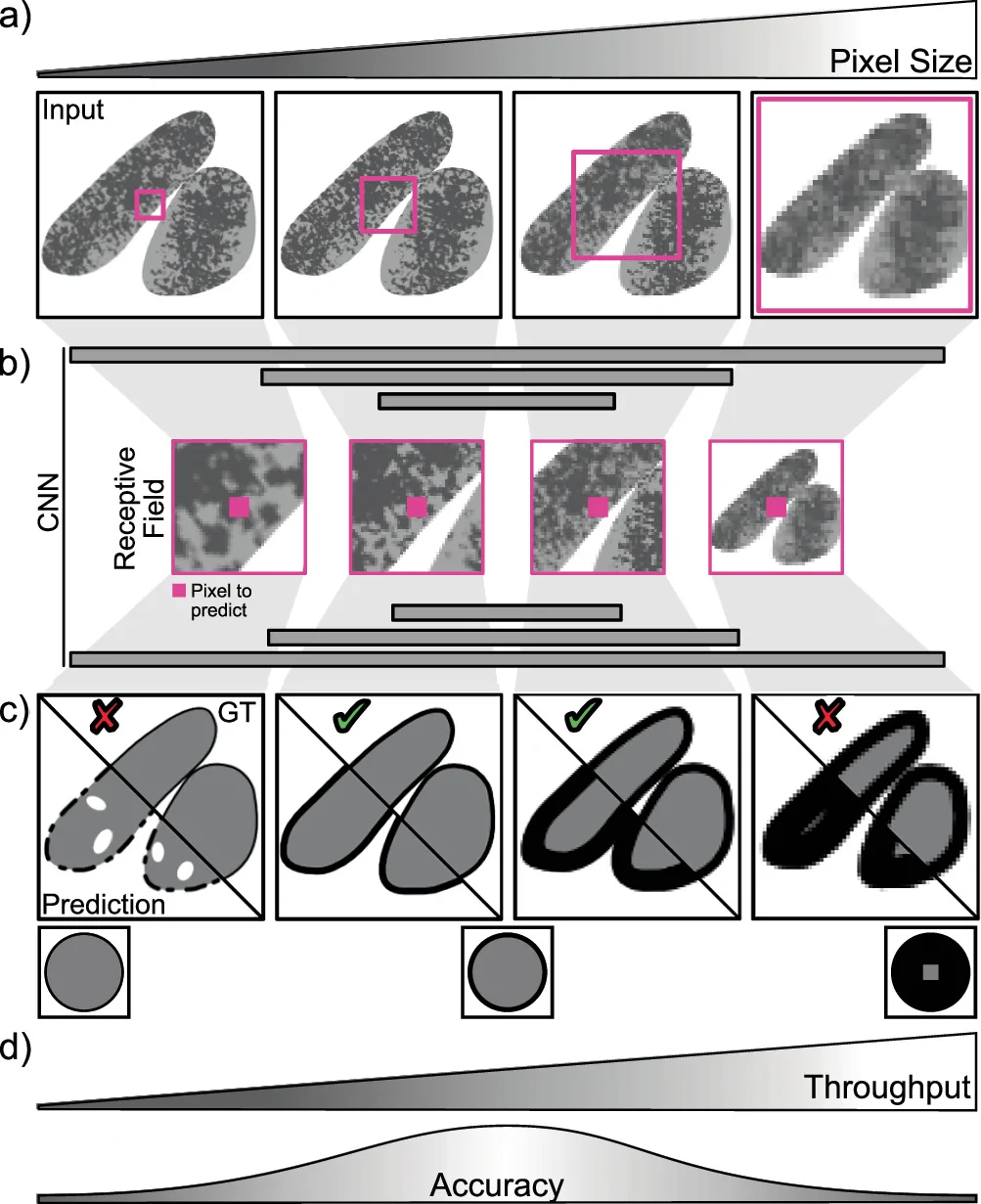
The Image Resolution Paradox. The counterintuitive relationship between image resolution and deep learning performance emerges from the mismatch between pixel size and the network’s receptive field. **a** Decreasing image resolution (increasing pixel size) causes pixelation and limits observable details, yet may improve contextual information. **b** The area observable within a convolutional neural network’s (CNN) receptive field (magenta squares) determines the context available for predicting the value of a single pixel. **c** Suboptimal pixel sizes lead to impaired predictions—either fragmented segmentations caused by non-continuous edges and inner pixels misclassified as background, referred to as false negatives (too high resolution) or over-generalisation at edges (too low resolution). The icons represent in order from left to right: 1) fragmented segmentations caused by non-continuous edges and inner pixels misclassified as background (i.e., false negatives), 2) accurate segmentation, and 3) over-generalization at edges. **d** Optimal image resolution for CNN-based processing depends on object size rather than maximal achievable microscopy resolution, and proper calibration can simultaneously enhance segmentation accuracy and experimental throughput.

We present this phenomenon as the image resolution paradox: While high-resolution microscopy captures more detail, this additional data may contribute minimally—or even negatively—to a certain analysis task while requiring substantially greater resources. This paradox is particularly evident in deep learning-based pipelines (Fig. 1). The limitation stems from convolutional neural network’s (CNN) receptive fields—the spatial region influencing a single output pixel. When image resolution exceeds the network’s architectural optimum, pixels lack sufficient contextual information, compromising the model’s performance. Practitioners often address this by downsampling the images during pre-processing. Others have already related the segmentation accuracy to the receptive field and contrast (i.e., SNR) in medical images^16^ and electron microscopy images^17^, and propose adapting the networks’ architecture to the data features accordingly^16^. Incorporating resolution and imaging considerations directly into the experimental design would enable acquisition at optimal resolutions from the outset, simultaneously increasing throughput and reducing computational demands.

While this paradox is familiar to computer vision experts, few tools explicitly account for it. Cellpose^18^ represents an exception, implementing automated re-sampling to optimise pixel-to-cell-diameter ratios. However, most users remain unaware of this critical consideration, leading to suboptimal application of deep learning methods—particularly with architectures like vanilla U-Net—and unnecessary prioritisation of high-resolution acquisition despite its potential drawbacks. Given the rise of user-friendly deep learning approaches and out-of-the-box foundation model-based solutions, practitioners would benefit from early systematic assessment routines.

**Fig. 2:**
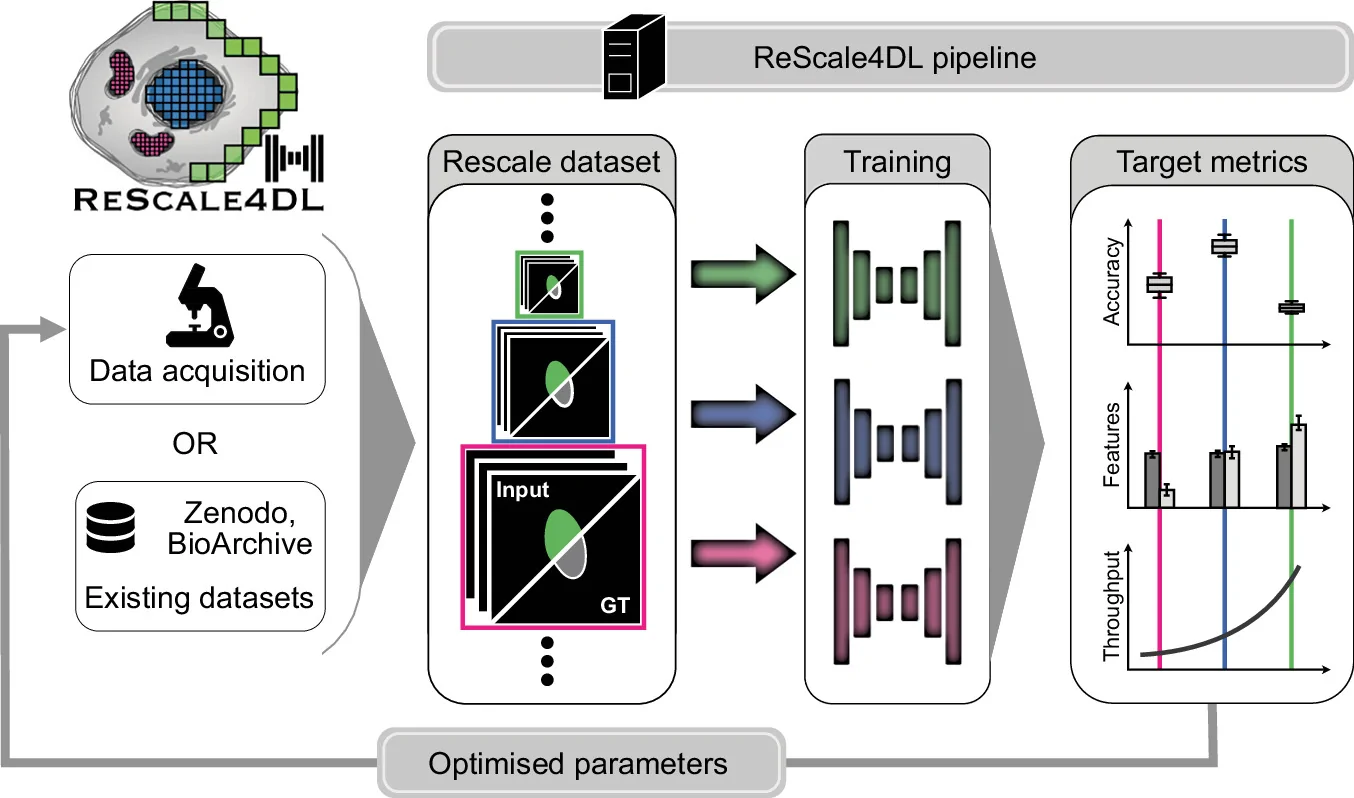
ReScale4DL pipeline. Researchers can optimise the pixel size for a chosen deep learning architecture using their newly acquired and annotated images or on existing datasets, which can be used as a starting point for the optimisation. A series of image rescaling factors is applied to the data, a deep learning model is trained for each rescaled dataset, and different accuracy metrics and features are computed. Once an ideal target metric is chosen, such as the accuracy of the image processing performance, the recovery of an image feature, or a good compromise between the throughput and accuracy, researchers can identify an adequate pixel size and launch their imaging experiments with optimised image resolution. The results presented in this manuscript were obtained by following the ReScale4DL pipeline.

In this study, we quantitatively demonstrate this paradox and its impact on segmentation performance in bioimage analysis. We systematically evaluate resolution effects on model accuracy using a canonical 2D U-Net^19^ for semantic segmentation and StarDist^9^ for instance segmentation. Our experiments employ diverse specimens, including high-magnification *Caenorhabditis elegans* (*C. elegans*)^20^ and brightfield *Escherichia coli* (*E. coli*)^21^ images. Additionally, we analyse *Staphylococcus aureus* (*S. aureus*) imaged with Structural Illumination Microscopy (SIM), demonstrating that critical phenotypic changes remain detectable even when reducing resolution by 16*×*, enabling proportional throughput improvements. Beyond these examples, we provide a generalisable framework, ReScale4DL (https://github.com/HenriquesLab/ReScale4DL), that researchers can implement to determine optimal pixel sizes for their specific contexts, maximising both computational efficiency and information extraction (Fig. 2).

## Results

### High resolution and segmentation accuracy: a counter-intuitive relationship

**Fig. 3:**
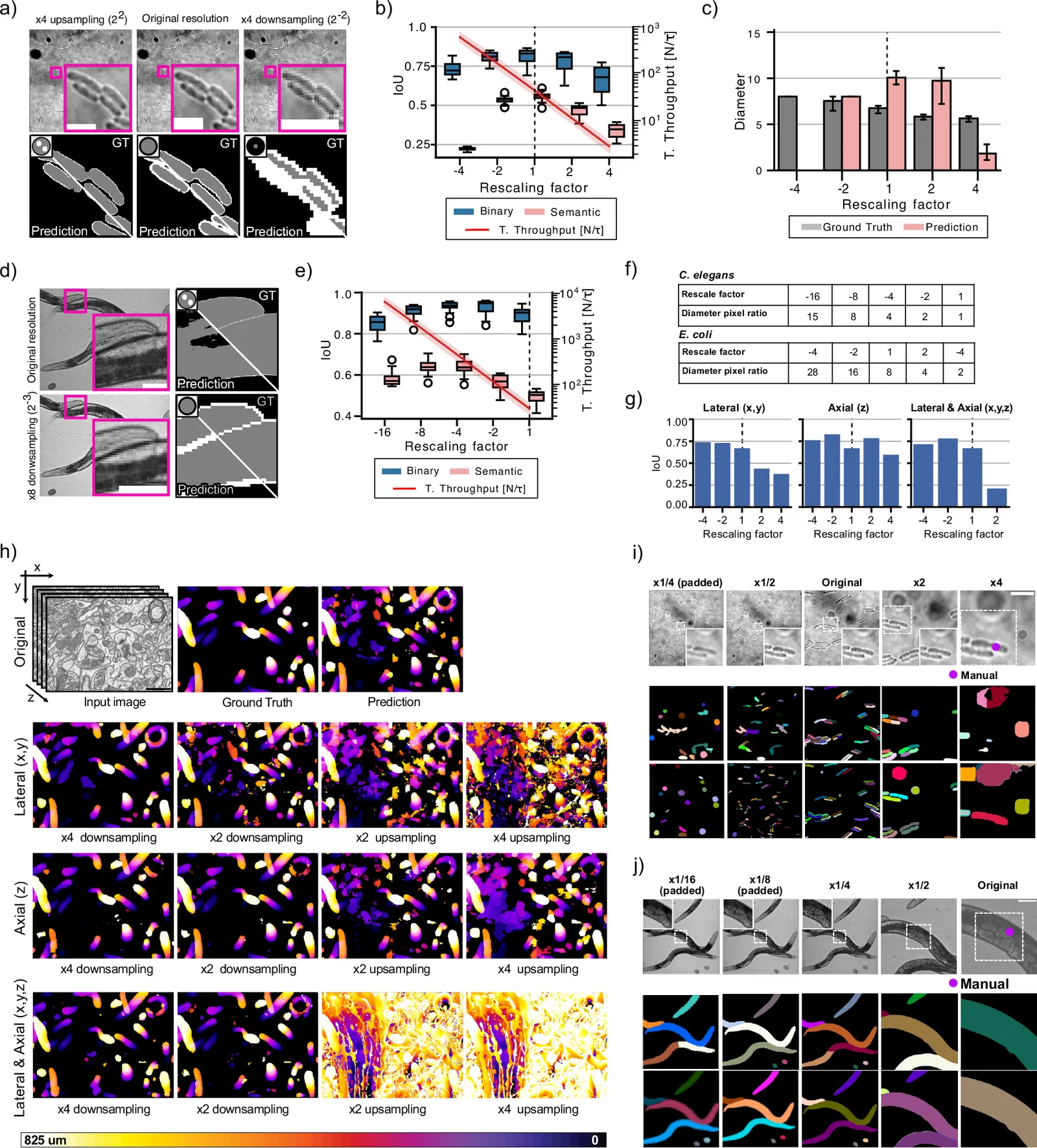
Impact of image resolution on deep learning segmentation accuracy. **a**–**f** Results of 2D U-Net models trained for semantic segmentation (inner area and boundary) of *E. coli* (**a**) and *C. elegans* (**d**) at varying simulated resolutions. **b** Intersection over Union (IoU) distributions (semantic and binary masks) across scaling factors for *E. coli*, ranging from *×*4 downsampling to *×*4 upsampling. IoU was calculated both for semantic segmentation (average IoU of edge and inner regions) and the binary masks resulting from considering the edge and inner regions. Purple line (log scale): theoretical throughput calculated as the number of objects observable by a unit of time (*τ*) given a certain resolution. **c** *E. coli* diameter distributions from U-Net segmentations versus ground truth. **e** Same as in (**b**), IoU for *C. elegans* segmentation at varying resolutions. **g** IoU on the test image volume of 3D U-Net mitochondria binary segmentation in transmission electron microscopy (TEM) at varying resolutions. **h** Results visualizations corresponding to z-colour-coded-projections at varying axial/lateral resolutions. **i**, **j** MicroSAM^22^ with a single point-wise prompting strategy was used to obtain the instance segmentation of *E. coli* (**i**) and *C. elegans* (**j**) example images. We pointed to the same object in each rescaled image and ran the automatic instance segmentation pipeline. All the images matched model input dimensions (512*×*512 pixels) to avoid internal tiling or image re-scaling. The images that had smaller dimensions were padded with zeros. Square boxes correspond to the same physical area on each image. Scale bars correspond to **a** 100, 25, and 10 pixels; **d** 100 and 25 pixels; **h** 200 pixels; **i**, **j** 50 *µm*. Circles in **b** and **e** correspond to data points outside the quartiles of the IoU distribution.

We trained a 2D U-Net architecture to semantically segment different cellular regions - inner area, edges, and background - using two distinct datasets: *C. elegans* captured with brightfield microscopy and *E. coli* imaged via phase contrast microscopy. For each dataset, we systematically rescaled the images to multiple resolutions and trained separate U-Net models to evaluate performance across these conditions (Fig. 2). Since segmentation accuracy depends on the relationship between image resolution and object size, we quantified the performance relative to the percentage of an object’s diameter covered by a single pixel. Our experiments revealed a counterintuitive relationship between resolution and segmentation quality (Fig. 3). Contrary to conventional expectations, high-resolution images often produced fragmented segmentation masks with unexpected discontinuities. In the *C. elegans* dataset, the inner masks produced at the original resolution contained spurious holes where pixels were misclassified as background (Fig. 3a). This occurred because the model lacked sufficient contextual information to confidently identify inner and outer objects’ areas and one-pixel width edges were under-represented for the given receptive field and resolution (see details in Supplementary Note 1, Supplementary Fig. 1). Namely, the Intersection over Union (IoU) of semantic segmentations decreased by approximately 40% compared to binary segmentation, which remained relatively high ( > 0.9) despite these deficiencies (Fig. 3b). Notably, downsampling the images improved accuracy metrics for both binary and semantic segmentation approaches, providing clear evidence that maximum resolution does not necessarily yield optimal segmentation results. The *E. coli* dataset analysis corroborated these findings (Fig. 3c-e).

To ensure consistent comparison, we maintained an identical U-Net architecture and hyperparameters across all experiments in Fig. 3, enabling direct assessment of resolution’s impact on model performance. Both datasets’ accuracy curves exhibited similar quadratic relationships between image resolution, object size, and IoU scores. For *C. elegans*, binary and semantic segmentation achieved peak IoU when pixels covered 2–4% and 4–8% of worm diameter, respectively (i.e., downsampling by a factor of 8 and 4), demonstrating that optimal resolution differed from the original microscopy acquisition (1% coverage).

Similarly, the *E. coli* dataset exhibited the highest IoU at comparable proportional resolutions: 4–16% diameter coverage per pixel for binary segmentation and 8–16% for semantic segmentation the original resolution and a downsampling by a factor of 2, respectively. This consistency across different biological specimens reveals a fundamental principle: our U-Net’s 48*×*48 pixel receptive field imposes practical constraints. Objects with diameters exceeding 25 pixels (corresponding to *<*4% diameter coverage per pixel) become problematic as their size exceeds the receptive field, while objects smaller than 6 pixels ( > 16% diameter coverage) risk information dilution and loss through the network’s operations. Namely, it is possible to recover optimal accuracy values when changing the U-Nets’ architecture to increase their receptive field and match the image resolution (see Supplementary Note 1, Supplementary Table 2 and Supplementary Fig. 1). Notwithstanding these updates, which require access to the code and programming skills, result in networks with a larger number of parameters, compromising computational requirements. As exemplified, for any given CNN architecture, there exists an optimal resolution that maximises segmentation accuracy–and importantly, this resolution is typically significantly lower than what human observers prefer, allowing imaging pipelines with substantially improved throughput. The paradox persists in emerging foundation models with transformer-based architectures despite their theoretical multi-scale capabilities. As demonstrated through MicroSAM^22^ foundation model evaluation, fixed context windows in pre-trained models remain susceptible to resolution mismatches (Fig. 3i–j and Supplementary Fig. 2).

### Optimising image resolution during acquisition improves segmentation accuracy and throughput

**Fig. 4:**
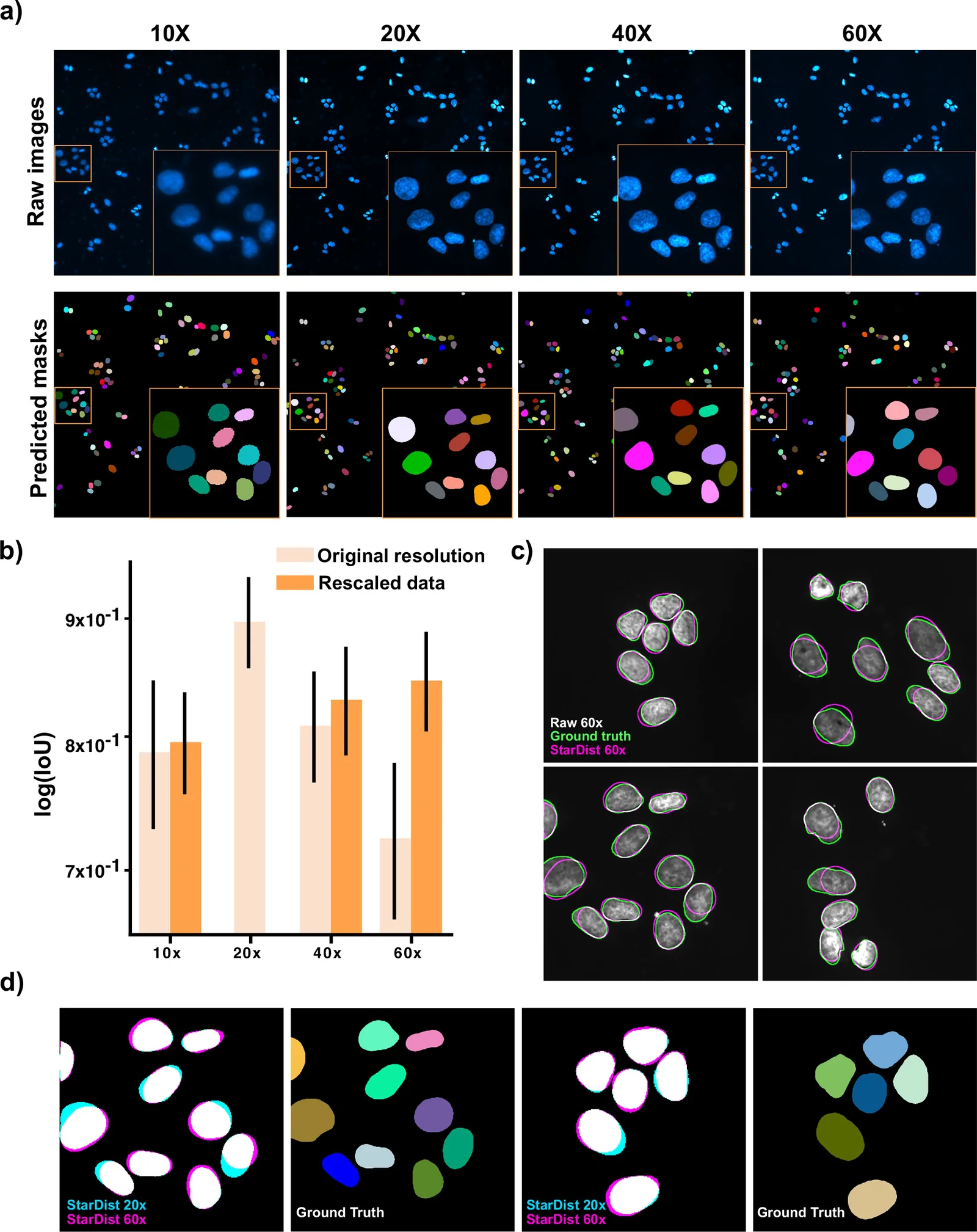
Segmentation accuracy of StarDist models across experimental imaging resolutions. DAPI-stained U2OS nuclei imaged at 10*×*, 20*×*, 40*×* and 60*×* objectives, with StarDist models trained per resolution. **a** Segmentation outputs from resolution-specific StarDist models. **b** Mean *±* s.d. Intersection over Union (IoU) for native-resolution models versus those trained on data rescaled to 20*×*-equivalent. **c** StarDist predictions on raw 60*×* data overlaid with Ground Truth. **d** Side-byside comparison of predictions from 20*×* and 60*×*-trained StarDist models.

To test the resolution paradox experimentally, U2OS nuclei were imaged with varying objectives (10*×*, 20*×*, 40*×*, and 60*×*) and manually annotated. This resulted in colocalised FOV at different resolutions. We then trained independent StarDist models to segment the same FOVs and assess the paradox (Fig. 4). In most cases, the accuracy was relatively high (IoU*>* 0.7; Fig. 4b), yet nuclei in images acquired at rather low resolution (20*×*) were more accurately segmented than those imaged at the highest resolution (60*×*). Optimal accuracy was recovered by rescaling all images to the pixel size equivalent to 20*×* resolution (Fig. 4b). The StarDist model trained with images at higher resolution (60*×*) estimates more rounded objects and over-segments large nuclei, likely due to insufficient contextual information for predicting varying nuclear shapes (Fig. 4c–d). Remarkably, 2D acquisition of the same FOV, considering an average displacement through the wheel, took 3.91, 9.51, 17.12, 36.48 s at 10*×*, 20*×*, 40*×* and 60*×*, respectively, implying that the acquisition at optimal quantitative conditions (20*×*) is also *∼*4*×* faster than at 60*×*. Notwithstanding, the FOV at 20*×* required a 3*×*3 tiling, while for 60*×* a tiling of 7*×*7 was needed, significantly increasing the exposure to fluorescence and therefore the induced photodamage in the overlapping regions.

### Optimising image resolution preserves phenotypic discrimination while maximising experimental throughput

**Fig. 5:**
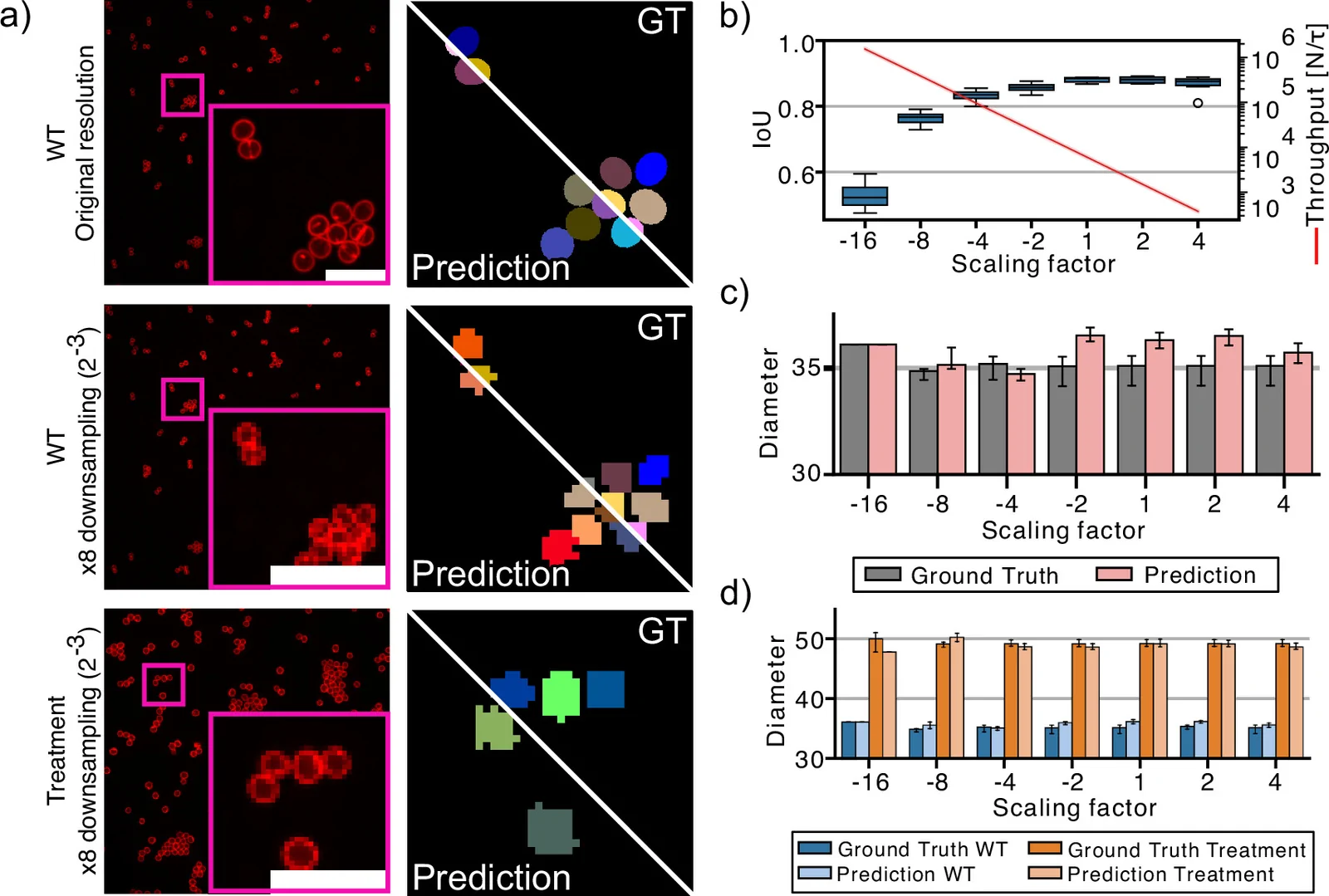
Deep learning-driven resolution adjustment enables phenotype distinction while increasing imaging throughput. *S. aureus* bacteria were treated with FtsZ inhibitor PC190723, which prevents cell division and results in larger bacteria phenotypes^23^. *S. aureus* (wild type (WT) and treated) were then imaged using Structured Illumination Microscopy (SIM) and segmented with StarDist. **a** Representative images of *S. aureus* with ground truth (GT) segmentation and StarDist results at the original resolution (WT) and after 2^3^-factor downsampling (both WT and treated conditions). Scale bars: 100, 25, and 25 pixels, respectively. **b** (Boxplots) Distribution of Intersection over Union (IoU) values of StarDist models trained and tested only with WT bacteria images across multiple resolution factors (upsampling: 2^*−*2^ to 2^0^; downsampling: 2^1^ to 2^4^), showing the relationship between segmentation accuracy and the percentage of bacterial diameter covered by a single pixel. (Purple line in logarithmic scale) Theoretical throughput was calculated as the number of objects observable by a unit of time (*τ*) given a certain resolution. *τ* is specific to each microscopy acquisition modality and preserved across the different resolutions. **c** Diameter distributions across resolution scales estimated from GT and the StarDist results for WT bacteria images in (**b**). **d** Comparison of the diameter distributions across various resolution scales for WT and treated bacteria. StarDist was trained with a mixed dataset containing WT and PC190723-treated *S. aureus* images in this case.

Building upon our findings, we demonstrate how optimising image resolution preserves segmentation performance while enhancing experimental throughput. We tested this approach using *S. aureus* bacteria treated with antibiotic PC190723 and imaged via SIM. PC190723, a FtsZ inhibitor, prevents cell division and produces enlarged bacteria^23^. To quantitatively assess this morphological effect, we employed StarDist—a deep learning approach optimised for instance segmentation of starconvex objects like round bacteria—to segment individual cells and measure diameter distributions (Fig. 5a). As in our previous experiments, IoU values across different resolution factors revealed an optimal range where segmentation accuracy peaks. Notably, even at substantially reduced resolutions, StarDist maintained robust performance: even when downsampling the images by a factor of 4 (11% of bacterial diameter covered by a single pixel), IoU values exceeded 0.8, and even with a factor of 8 (23% coverage), IoU remained above 0.75 (Fig. 5b and Supplementary Note 3). Analysis of wild-type (WT) diameter distributions confirmed that measurements derived from ground truth and StarDist predictions remained consistent across resolutions. At higher resolutions (scaling factors > *−*2), there is a minimal difference between the ground truth and predicted masks resulting from StarDist models segmenting the outer region of the cell (see Supplementary Fig. 3). At lower resolutions (scaling factors <*−*4), this difference disappears with objects being reduced to just a few pixels in width (Fig. 5c). Most importantly, when training a StarDist model with WT and antibiotic-treated mixed bacteria populations, the diameter differences remained distinguishable across all sampling ratios, demonstrating that phenotypic discrimination is preserved even at significantly lower resolutions than typically employed (Fig. 5d). Thus, by strategically reducing acquisition resolution, researchers can substantially increase imaging throughput and decrease acquisition times (Figs. 3b, 3e, and 5b), enabling more comprehensive dataset collection for statistically robust experimental analyses.

## Discussion

Our systematic investigation of the resolution paradox in deep learning-driven bioimage analysis reveals a fundamental tradeoff between spatial resolution and computational performance. Our results demonstrate that popular methods like U-Net and StarDist (which often use a U-Net architecture) achieve optimal accuracy when image resolution is calibrated to match the network’s receptive field with biological object dimensions, rather than maximising resolution. This finding challenges conventional microscopy practices while offering significant practical benefits for experimental workflows.

The paradox emerges from fundamental neural network characteristics. When object features extend beyond the receptive field (high-resolution), networks lack sufficient context to predict boundaries consistently, resulting in fragmented masks with spurious discontinuities, in line with the results from previous works^16,17^. Conversely, excessively downsampled images (low-resolution) compress critical morphological features beyond recognition. Our quantitative analysis reveals a predictable quadratic relationship between object diameter coverage per pixel and segmentation accuracy, with optimal performance for the 2D U-Net consistently occurring when pixels cover 4-16% of object diameter—irrespective of the type of specimen or imaging modality. This optimal range represents a “sweet spot” where networks balance local detail with global context. We further test our resolution paradox using colocalised acquisitions at different optical resolutions and show an equivalent quadratic relationship between the network performance and the image resolution. Remarkably, the paradox persists in emerging foundation models with transformer-based architectures despite their theoretical multi-scale capabilities. Despite the increased complexity of these novel architectures, the growing ecosystem of interactive tools like BioImage Model Zoo^6^, MicroSAM^22^ and DinoSim^24^ creates opportunities for rapid empirical optimisation, turning this challenge into an accessible experimental design parameter.

Most significantly, we demonstrated that models trained on optimally downsampled images maintain phenotypic discrimination capabilities. In the *S. aureus* antibiotic response experiment, critical morphological differences remained detectable even at 16*×* lower resolution than commonly used (Fig. 5). These findings together have profound implications for experimental design: researchers can substantially increase imaging throughput, speed up image acquisition, increase temporal resolution, reduce indirect photobleaching and phototoxicity resulting from tiling, minimise data storage requirements, and accelerate analysis pipelines—all without compromising biological insights^25,26^.

Although these principles are established in computer vision communities, they remain underappreciated in life sciences, where imaging protocols continue to emphasise the maximum achievable resolution regardless of computational analysis requirements. Our work provides a practical framework for biologists to determine optimal resolution parameters for their specific experimental contexts. By conducting preliminary resolution calibration studies before full-scale data acquisition, researchers can design more efficient imaging protocols that balance biological information content with computational performance.

The proposed image rescaling with ReScale4DL is a simple, architecture-agnostic, broadly accessible strategy that complements, rather than replaces, more advanced receptive-field engineering. Importantly, ReScale4DL proposes a way to optimise imaging conditions considering the accuracy of image segmentation, which can be an intermediate step of an image analysis pipeline. For example, it can precede a cell tracking step, for which acquiring images at lower resolution can also be beneficial due to increased acquisition speed and field of view. Likewise, segmentation is often an intermediate step for further quantification within an area and for which a higher-resolution image is more convenient, such as measuring single-cell protein concentration or organelle distribution. In these cases, an optimal image resolution for the segmentation task can be achieved post-acquisition by using the ReScale4DL rescaling schema. Yet, researchers should always consider the Nyquist sample principle when deciding on their minimal imaging resolution to ensure that the features to be measured are adequately sampled. Democratising AI in bioimaging requires deeper integration of data physics with model architectures. Current trends favour pre-trained “smart” solutions over adaptable systems, risking suboptimal application to novel experimental contexts. Future developments should prioritise dynamic acquisition systems that adjust resolution in real-time based on ongoing analysis, coupled with neural architectures explicitly encoding multi-scale relationships. By embedding these considerations into standard workflows, the field can achieve reproducible, resource-efficient microscopy pipelines that balance information content with computational sustainability.

## Methods

### Biological image datasets

Publicly available high-resolution *C. elegans* minimum projection brightfield microscopy images and instance segmentation masks were obtained from ref.^20^. Publicly available *E. coli* brightfield microscopy images and instance segmentation masks dataset was obtained from^21^, which is available on Zenodo 10.5281/zenodo.5550934. In-house acquired super-resolution *S. aureus* cell wall structured illumination microscopy (SIM) fluorescence images and annotated instance segmentation masks dataset available on Zenodo 10.5281/zenodo.15169017.

#### Hoechst nuclear staining of U2OS fixed cells

U2OS cells were seeded at a density of 1*×*10^4^ cells per well in Cellvis 8-well glass-bottom chambers using 200–300 µL of complete culture medium per well and incubated overnight at 37^*◦*^C in a humidified atmosphere containing 5% CO_2_.

After 24 h, all buffers were pre-warmed to 37^*◦*^C prior to use. Culture medium was aspirated and cells were fixed for 10 min in CB buffer containing 4% paraformaldehyde (PFA) and 1% glutaraldehyde (GA). Residual aldehydes were quenched by incubation with freshly prepared 0.5% sodium borohydride (NaBH_4_) in PBS for 7 min. Cells were subsequently washed sequentially in PBS for 1 min, 5 min, and 10 min.

Nuclei were stained with Hoechst 33342 at a final concentration of 1 µg/mL using 200 µL staining solution per well for 10–15 min in the dark. Samples were protected from light throughout the staining procedure. Following staining, samples were washed twice with PBS for 5 min each and post-fixed with 4% PFA for 10 min followed by three PBS washes. Imaging was performed directly in the chamber using fluorescence microscopy on a Nikon Ti2 microscope at varying magnifications (10*×*,20*×*,60*×*,40*×* objectives).

### Dataset re-sampling

ReScale4DL Python library (https://github.com/HenriquesLab/ReScale4DL) was used to resize raw images and their respective masks. For *C. elegans* and *E. coli*, the instance masks were first rescaled and then the edges and inner side were estimated using the ImageJ macro available in DeepBacs GitHub repository^21^ (https://github.com/HenriquesLab/DeepBacs/). Upsampling was computed with Catmull-Rom interpolation and nearest-neighbor interpolation methods for the raw and the mask images, respectively (all available within the NanoPyx Python package^27^). Downsampling was computed with binning and transform.rescale from scikit-image for the raw images and the masks, respectively. *C. elegans* dataset was downsampled by factors 2, 2^2^, 2^3^, and 2^4^. *E. coli* was upsampled by factors 2 and 2^2^ and downsampled by factors 2 and 2^2^. *S. aureus* was upsampled by factors 2 and 2^2^ and downsampled by factors 2, 2^2^, 2^3^, and 2^4^.

### Segmentation networks

The 2D Multilabel U-Net and 2D StarDist^9^ models were trained using the respective ZeroCostDL4Mic^2^ notebooks in local GPU-equipped workstations with DL4MicEverywhere^11^ and the hyperparameter configuration specified as in Supplementary Table 1 in Supplementary Note 1.

### Evaluation

The Intersection over Union (IoU) scores visualised in Figs. 3 and 5 were calculated for the results of all the models specified in Supplementary Table 1 in Supplementary Note 1, and for the binary segmentation (*C. elegans* and *E. coli*), semantic segmentation (*C. elegans* and *E. coli*) and instance segmentation (*S. aureus*) results. For each object *j* in an image *i*, the diameter *D*_*ij*_ of non-rounded shapes (*C. elegans* and *E. coli* datasets) was computed as the median of the Euclidean Distance Transform (EDT) values contained in the object’s skeleton. For *S. aureus* dataset, *D*_*ij*_ of each bacteria was estimated by solving the equation of the circle area, given as

1 $${D}_{{ij}}=2 \cdot \sqrt{\frac{{A}_{{ij}}}{\pi }}$$

where *A*_*ij*_ is the area of the object *j* in the image *i*, measured as the sum of all the pixels in the object. To compute the average portion of diameter covered by one pixel, we first computed the average diameter for each scaling factor, *D*_*s*_ as

2 $${D}_{s}=\frac{1}{N}{\sum }_{i=1}^{N}\left(\frac{1}{{O}_{i}}{\sum }_{j=1}^{{O}_{i}}{D}_{{ij}}\right),\,s\in \left[{2}^{{{{\rm{{Z}}}}}_{[-2,4]}}\right]$$

where *O*_*i*_ is the total number of objects in the image *i*, *N* is the total number of images in the dataset and *s* is the rescaling factor. Then, *D*_*s*_ was inverted and multiplied by 100 to compute the portion of diameter covered by one pixel.

To measure the throughput achievable for each rescaling factor *s*, we first estimated the average area of each object in an image (*A*_*s*_) in pixels. Then, we consider the size of the achievable field of view (FOV) for a camera as the area in pixels of the images in the original dataset (*CAM*_*FOV*_). Considering the throughput as the amount of information recordable per unit of time, we computed the throughput as

3 $${{TH}}_{s}=\frac{{{CAM}}_{{FOV}}}{{A}_{s}}\times \frac{1}{\tau },\,s\in \left[{2}^{{{{\rm{{Z}}}}}_{[-2,4]}}\right]$$

where *τ* is the time required to acquire a *FOV*. In other words, *TH*_*s*_ provides an estimate of the number of objects that can be captured by a unit of time, yet, using Eq. 3 with preliminary information about the sample’s morphology and microscopy camera features, it is possible to compute this value easily.

### Reporting summary

Further information on research design is available in the Nature Portfolio Reporting Summary linked to this article.

## Supplementary information

Supplementary Information

Reporting Summary

Transparent Peer Review file

## Data Availability

W.T. and antibiotic-treated super-resolution *S. aureus* cell wall SIM images together with instance segmentation annotations are available on Zenodo https://zenodo.org/records/15169018. Manually annotated U2OS images are available on Zenodo (https://zenodo.org/uploads/20041089). All the trained models are also available in Zenodo (https://zenodo.org/records/20040567). These models can all be loaded, tested and retrained through the respective notebooks in the DL4MicEverywhere platform^11^.

## Code availability

The code for image rescaling and metric computation is available at https://github.com/ HenriquesLab/ReScale4DL under version ReScale4DL—0.2.2. The *rescale*4*dl* Python package is also made available through *PyPI* and can be installed by running a *pip install* command. The notebooks to train, test and deploy inference are available through the DL4MicEvrywhere (https://github.com/HenriquesLab/DL4MicEverywhere) platform.
