# Supplementary material for: ReScale4DL: balancing pixel and contextual information for enhanced bioimage segmentation
Source: Nat Commun. 2026 Sep 18;17:10942. doi: 10.1038/s41467-026-77930-1 (PMC13680607; doi:10.1038/s41467-026-77930-1)
Supplement: Supplementary file 1 [file 41467_2026_77930_MOESM1_ESM.pdf]

Supplementary Material
ReScale4DL: Balancing Pixel and Contextual Information for Enhanced Bioimage Segmentation

Mariana G. Ferreira[1], **Bruno M. Saraiva**[1], **António D. Brito**[1,2], **Mariana G. Pinho**[2], **Ricardo Henriques**[1,3,✉], **and Estibaliz Gómez-de-Mariscal**[1,4,5,✉]

[1]AI-driven Optical Biology Laboratory, Instituto de Tecnologia Química e Biológica António Xavier, Universidade Nova de Lisboa, Oeiras, Portugal
[2]Bacterial Cell Biology Laboratory, Instituto de Tecnologia Química e Biológica António Xavier, Universidade Nova de Lisboa, Oeiras, Portugal
[3]UCL Laboratory for Molecular Cell Biology, University College London, London, United Kingdom
[4]NOVA Institute for Medical Systems Biology (NIMSB), Universidade Nova de Lisboa, Lisboa, Portugal
[5]iNOVA4Health, Programme in Translational Medicine, Lisboa, Portugal

Supplementary Note 1: Deep learning model training hyperparameters

C. elegans		*E. coli*		*S. aureus*	
Network	2D U-Net	Network	2D U-Net	Network	2D StarDist
Epochs	1000	Epochs	1000	Epochs	1000
Patch size (per resizing factor)	512 (x$2^{-2,-1,0}$)<br>256 (x2)<br>128 (x2^2)<br>64 (x2^3)<br>32 (x2^4)	Patch size (per resizing factor)	512 (x$2^{-2,-1,0}$)<br>256 (x2)<br>128 (x2^2)	Patch size (per resizing factor)	2048 (x$2^{-2,-1,0}$)<br>1184 (x2)<br>576 (x2^2)<br>272 (x2^3)<br>128 (x2^4)
Batch size	5	Batch size	5	Batch size	2
Validation [%]	10	Validation [%]	10	Validation [%]	10
Learning rate	0.0001	Learning rate	0.0001	Learning rate	0.0003
Pooling steps	2	Pooling steps	2	Grid parameter	2
Minimal fraction	0.05	Minimal fraction	0.05	Number of rays	32

Supplementary Table 1. The network architecture and the training parameters for each dataset were preserved. The patch size for the input during training was dynamically changed with the image resizing to maintain the same field of view, except for the upsampling due to limited GPU capabilities. The values $2^{-2,-1}$ correspond to image upsamplings, 2^0 is the original image resolution and $2^{1,\ldots,4}$ correspond to image downsampling.

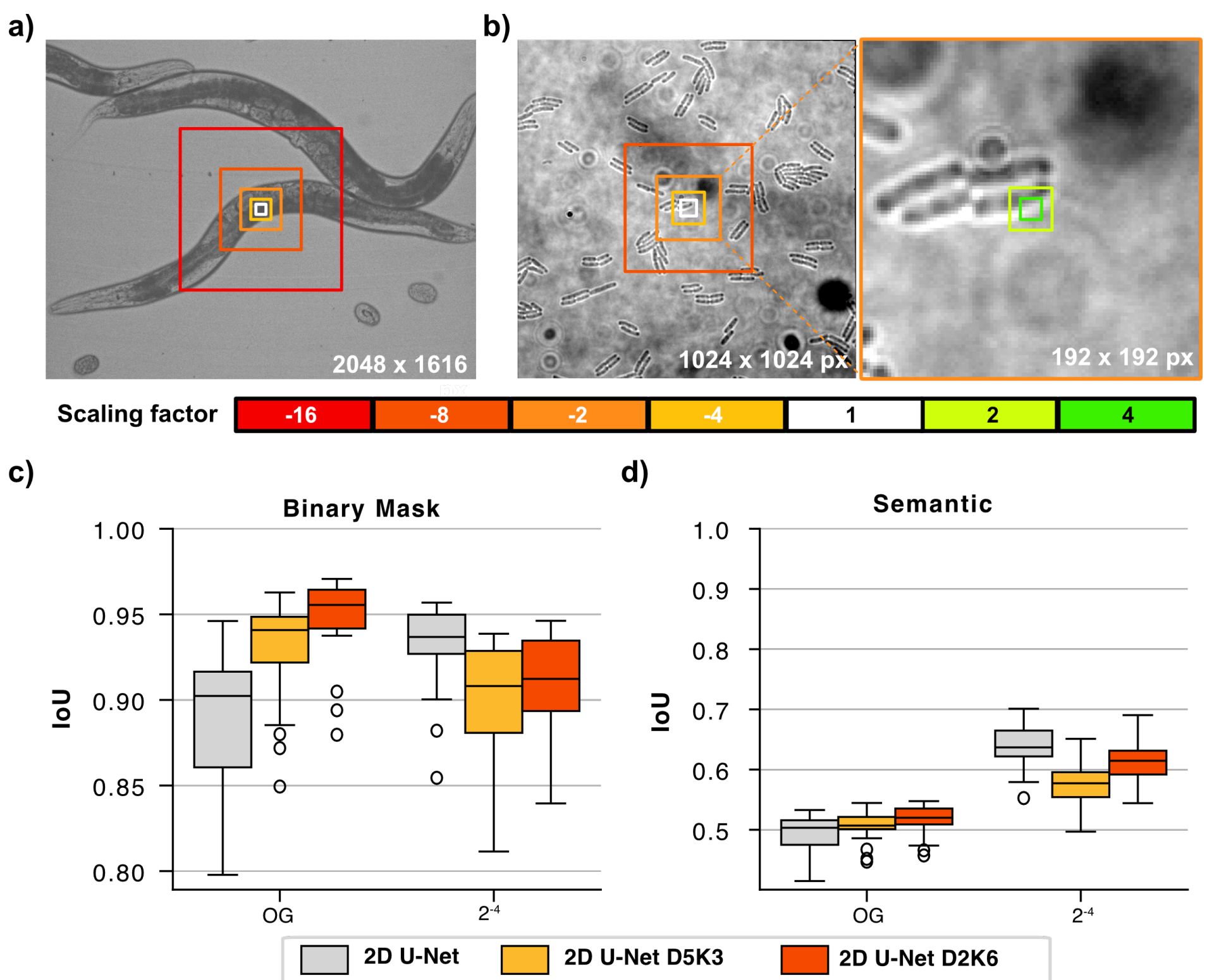

Supplementary Fig. 1. Receptive field with respect to the scaling factors A 2D U-Net with two pooling layers and a receptive field of 48×48 pixels was used to process a) *C. elegans* and b) *E. coli* datasets. When rescaling the images, the relationship between the receptive field and the image changes, being larger when downsampling the images and smaller when upsampling. c-d) U-Net architectures with two pooling layers and varying receptive fields were trained to segment *C. elegans* dataset at the original resolution and downsampled by a factor of 2^4 (a). Starting from the baseline U-Net with two pooling operations, we increased the effective receptive field by modifying only the convolutional kernel size and the dilation/stride parameters, thereby approximating the receptive field obtained when downsampling the input images (Supplementary Table 2). Increasing the receptive field can recover segmentation performance comparable to image downsampling. The total number of images analysed is $n = 20$. Boxplots display the mean, 25% and 75% quartile values.

Name	Kernel size	Stride	Receptive field	Trainable parameters	Equivalent downsampling
2D U-Net	3×3	1	48×48 pixels	1,863,375 (7.11 MB)	1
2D U-Net D5K3	3×3	5	208×208 pixels	1,863,375 (7.11 MB)	2^2
2D U-Net D2K6	6×6	2	211×211 pixels	6,957,519 (26.54 MB)	2^2

Supplementary Table 2. Comparison of U-Net architectures with modified receptive fields (Supplementary Fig. 1c-d).

Supplementary Note 2: Optimising the pixel size for foundation models

We performed instance segmentation of *E. coli*, *C. elegans*, and *S. aureus* samples using MicroSAM (1) software, with the same rescaling factors used for the Figs. 3 and 5. For simplicity, we used their 2D Annotator plugin in Napari to deploy interactive segmentation with a single-point prompt-based approach, followed by the automatic instance segmentation. Rescaled images were individually segmented by manually pointing to the same structural landmark each time. For all experiments, we chose the Vision Transformer (ViT) Large backbone architecture, a fine-tuned version of the original Segment Anything Model (SAM) (2) for cellular and nuclear segmentation in light microscopy (`ViT-l-lm`). Importantly, the segmentation results provided here may not be optimal and only aim to show the variability of the model according to the image resolution under the same conditions. Indeed, the results could be improved by changing the prompt or by increasing the number.

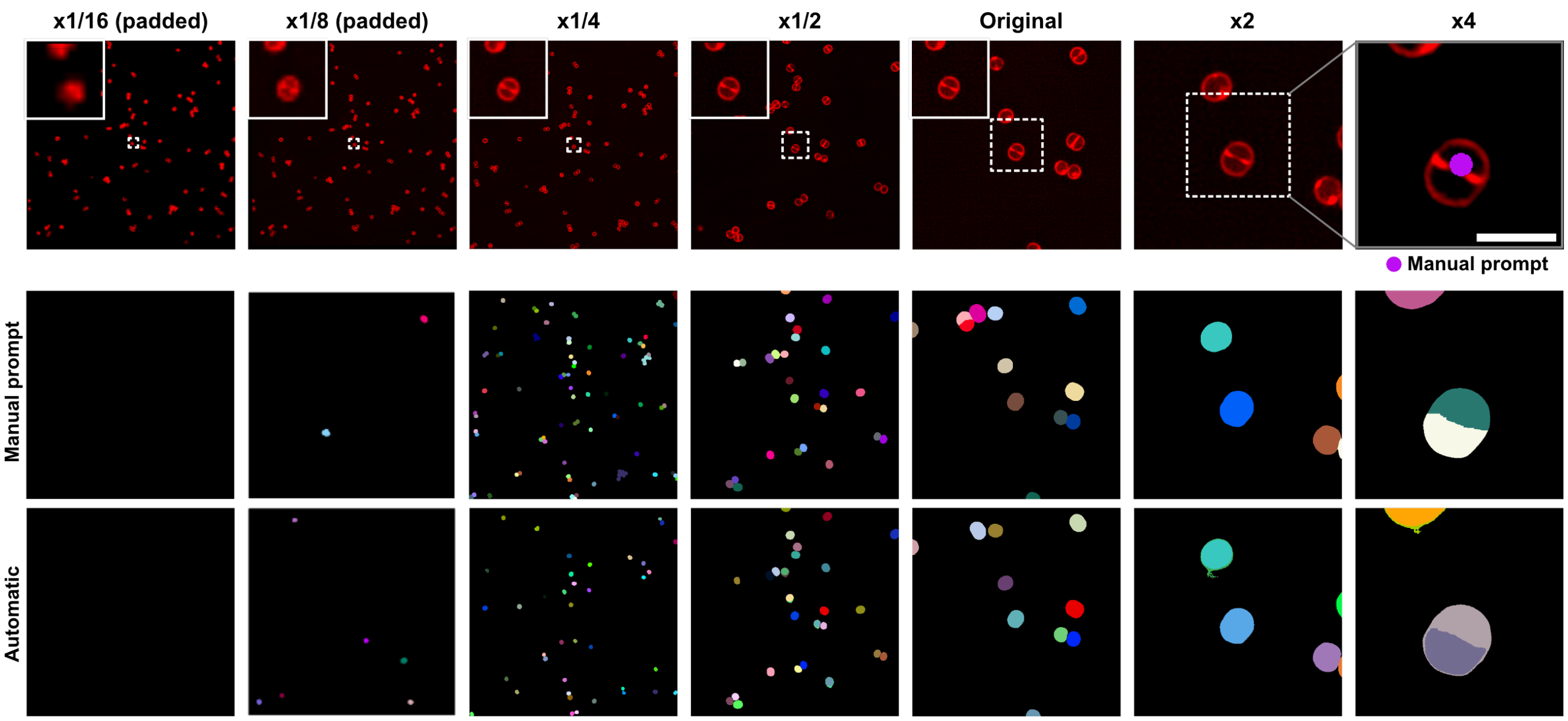

Supplementary Fig. 2. Segmentation results of Segment Anything for Microscopy on rescaled images using a single point prompting. MicroSAM (1) was used to obtain the instance segmentation of a *S. aureus* example image. For simplicity, we followed a single point-wise prompting strategy, pointing to the same object on each rescaled image (*i.e.*, the manual prompt was the same on each panel), and ran the automatic instance segmentation pipeline. All the images matched model input dimensions (512×512 pixels) to avoid internal tiling or image re-scaling. The images that had smaller dimensions were padded with zeros. Scale bars correspond to $25\ \mu m$. Square boxes correspond to the same physical area on each image.

Supplementary Note 3: Pixel size and diameter relationship for *S. aureus*

S. aureus	Pixel Diameter [%]	
Rescaling factor	Wildtype	Treatment
2^2	0.71	0.5
2	1.41	1.0
**1**	**2.84**	**2.1**
2^{-1}	5.67	4.1
2^{-2}	11.35	8.2
2^{-3}	22.73	16.5
2^{-4}	45.20	32.8

Supplementary Table 3. Rescaling factors for *S. aureus*. The rescaling factor indicates data upsampling or downsampling, depending on whether it is higher or lower than one, respectively. Pixel diameter $[\%]$ refers to the proportion of the diameter covered by one pixel for the wild-type and treated bacteria samples.

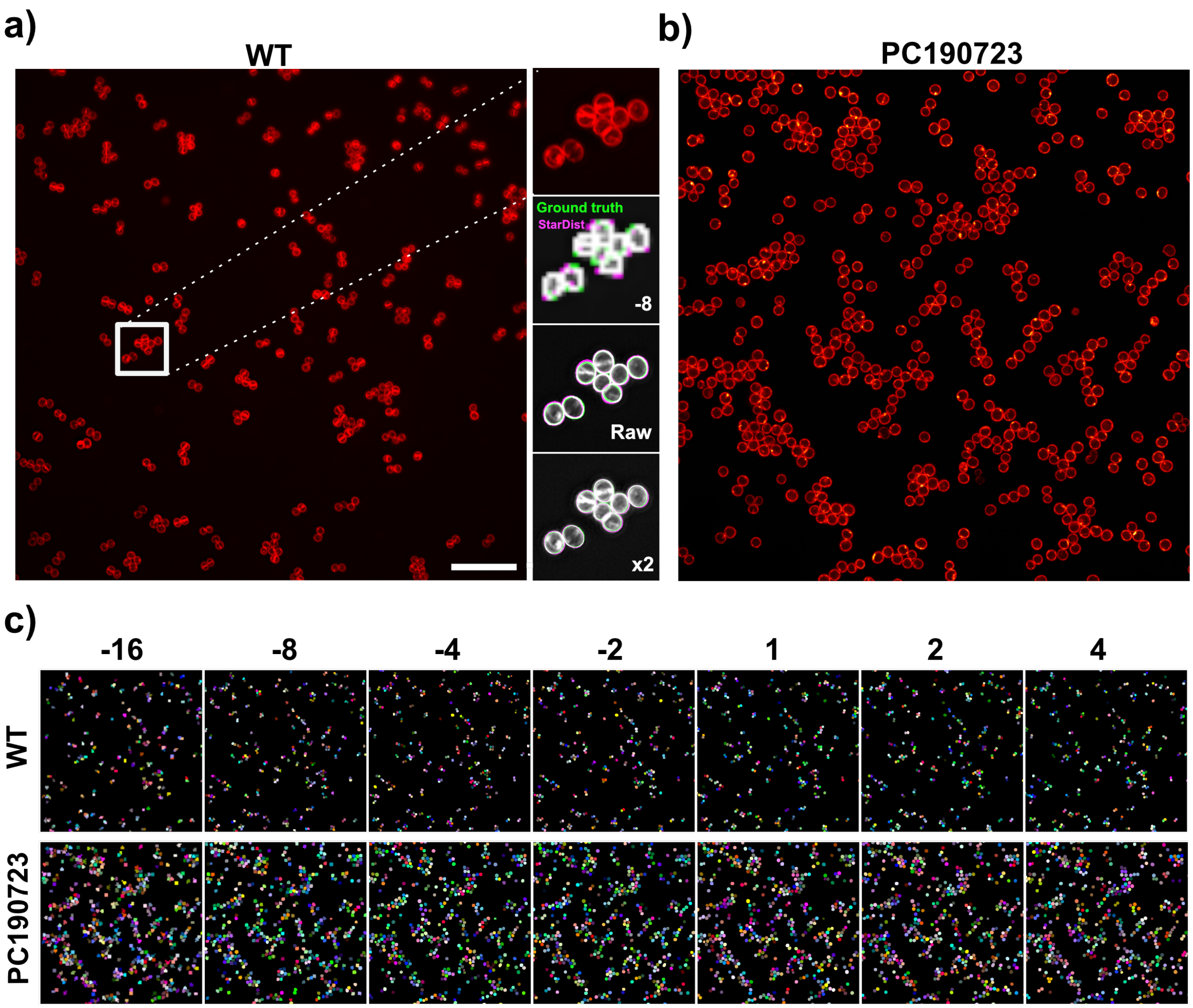

Supplementary Fig. 3. Visual examples of *S. aureus* segmentation results a) Original image of wild type (WT) *S. aureus* and corresponding StarDist segmentation results (magenta) and ground truth (green) at different scaling factors. At higher resolutions (upsampling by a factor of 2), the StarDist model segments bacterial cells over a wider area than the manually labelled one. This difference decreases with lower resolutions (downsampling by a factor of 8). b) Original image of treated *S. aureus*. Scale bar $10\mu m$ in a) and b). c) Segmentation results for the FOVs in a) and b) at scaling factors ranging from -16 until 4.

Supplementary Note 4: Statistical comparison

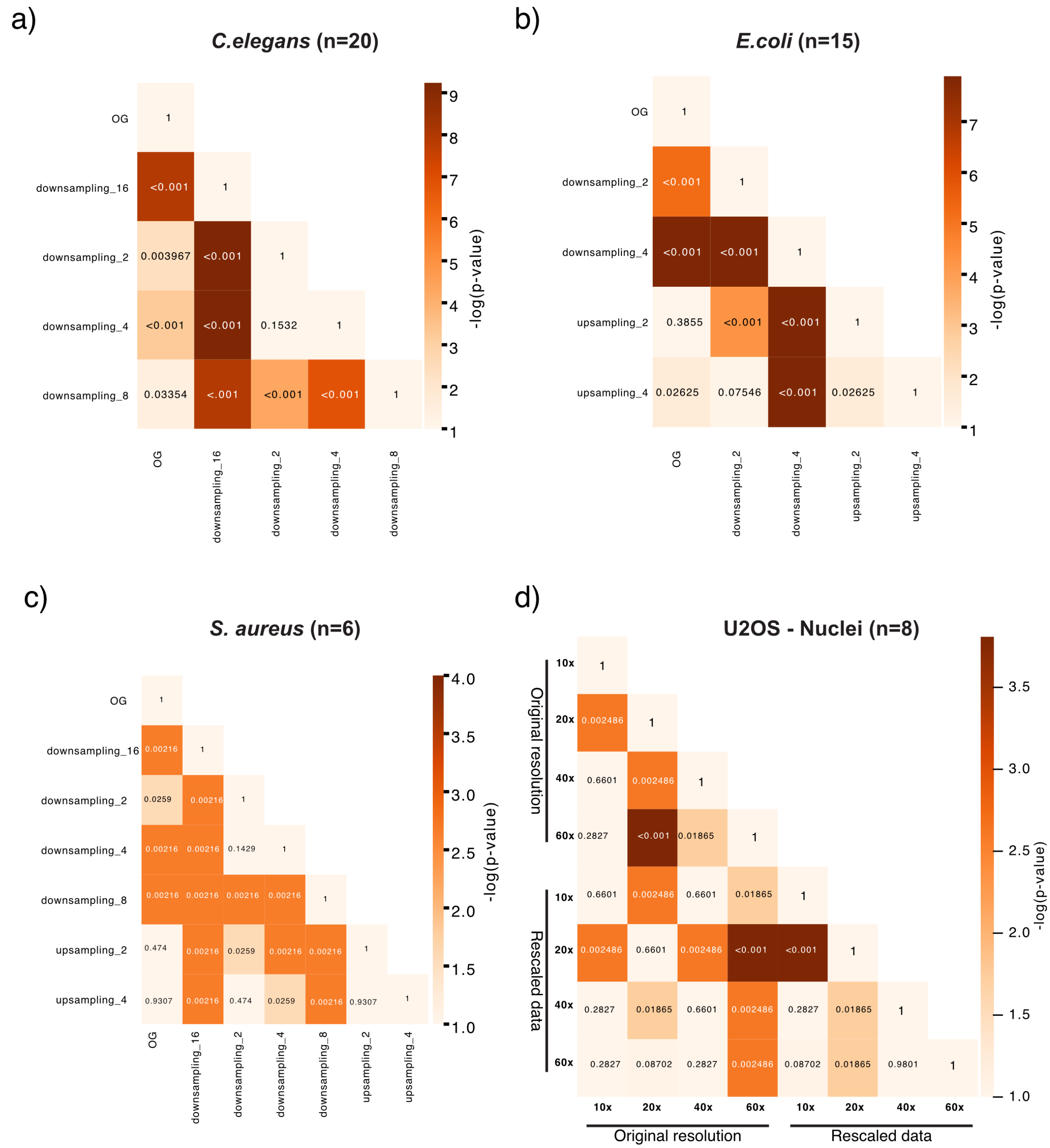

Supplementary Fig. 4. Kolmogorov-Smirnov statistical hypothesis tests. IoU mean value distribution for the (a-b) semantic segmentation of *C. elegans* (Supplementary Fig. 3e) and *E. coli* (Supplementary Fig. 3b), and (c-d) the instance segmentation of *S. aureus* (Supplementary Fig. 5b) and U2OS nuclei (Supplementary Fig. 4b) were compared using the Kolmogorov-Smirnov statistical hypothesis test. For each image an IoU value is obtained and the n value corresponds to the total number of images. Being an instance segmentation task, in c) and d), the IoU of each cell in the image is calculated and the average IoU of all the cells in the image is the one considered for the statistical test.

References

1. Anwai Archit, Luca Freckmann, Sushmita Nair, Nabeel Khalid, Paul Hilt, Vikas Rajashekar, Marei Freitag, Carolin Teuber, Genevieve Buckley, Sebastian von Haaren, Sagnik Gupta, Andreas Dengel, Sheraz Ahmed, and Constantin Pape. Segment Anything for Microscopy. *Nature Methods*, 22(3):579–591, March 2025. ISSN 1548-7105. doi: 10.1038/s41592-024-02580-4. Publisher: Nature Publishing Group.
2. Alexander Kirillov, Eric Mintun, Nikhila Ravi, Hanzi Mao, Chloe Rolland, Laura Gustafson, Tete Xiao, Spencer Whitehead, Alexander C. Berg, Wan-Yen Lo, Piotr Dollár, and Ross Girshick. Segment Anything. April 2023.
