# Supplementary material for: ReScale4DL: balancing pixel and contextual information for enhanced bioimage segmentation
Source: Nat Commun. 2026 Sep 18;17:10942. doi: 10.1038/s41467-026-77930-1 (PMC13680607; doi:10.1038/s41467-026-77930-1)
Supplement: Supplementary file 3 [file 41467_2026_77930_MOESM3_ESM.pdf]

nature portfolio

Peer Review File

ReScale4DL: Balancing Pixel and Contextual Information for Enhanced Bioimage Segmentation

Corresponding Author: Professor Ricardo Henriques

This file contains all reviewer reports in order by version, followed by all author rebuttals in order by version.

Version 0:

Reviewer comments:

Reviewer #1

(Remarks to the Author)
Short Summary
Ferreira et al. introduce ReScale4DL, a pipeline that systematically resamples annotated light-microscopy images at multiple scales and retrains segmentation models (U-Net, StarDist) on each version. By measuring Intersection-over-Union (IoU), they pinpoint an optimal pixel-to-object-diameter ratio (~ 4 %–16 %) that maximizes accuracy while dramatically boosting throughput. Tested on 2D brightfield, phase-contrast, and SIM datasets, imaging at this "sweet spot" yields smoother masks and enables 15x–40x faster data collection without sacrificing performance. The key insight is that highest resolution isn't always best—matching pixel size to the model's effective receptive field (~ 4 %–16 % of object diameter) optimizes both segmentation and experimental efficiency.

Recommendation
This reviewer finds the study suitable for publication. Consideration of the feedback below would strengthen the study.

Major Feedback
• It would be helpful to include key training hyperparameters (learning rate, batch size, number of epochs). This reviewer looked through the paper and the GitHub repo but couldn't readily find them, and the referenced table ("Table ??") in the GitHub repo readme appears missing.
• Similarly, adding sample sizes and a simple statistical test to the IoU results would make it clearer how robust the differences are. I couldn't find that information in the paper or repo.
• All experiments use 2D light-microscopy images. Testing on a 3D EM dataset (e.g., FIB-SEM) would show whether the 4 %–16 % pixel-diameter guideline holds for volumetric data. Because all tests are on light microscopy, including an EM example would confirm that the "sweet spot" generalizes to higher-resolution modalities.

Minor Feedback
• Terminology around "image resolution" vs. "pixel size" is used inconsistently; a brief early definition distinguishing these terms would improve clarity.
• In the Discussion (right after mentioning MicroSAM, reference 23), there are placeholders "Note ??, Fig. ??" that need updating before publication.
• The throughput curves (Figures 3b, 3d, and 4b) are plotted on a logarithmic axis but do not include clear axis labels or units (e.g., "objects per second"), which could confuse readers.
• Some legends use both "x" notation (e.g., "2x downsampling") and percentage coverage interchangeably; standardizing to one style would be preferable.
• Color choices in plots (especially the purple throughput curve) may be hard to distinguish in grayscale or for color-blind readers; adding dashed lines or markers would help.
• Small typos and formatting inconsistencies appear (e.g., extra line breaks splitting sentences in the Methods); a final proofread would catch these.
• While code availability is noted, version tags or commit hashes for the ReScale4DL repository are missing, which would aid reproducibility.

Of Interest, but Not Essential
• The throughput gains are modeled, but adding a brief real-world example (e.g., a live-cell time course) would illustrate how downsampling impacts photobleaching or frame rates in practice.
• Since downsampling only by powers of two might skip "in-between" scales (like ~10 % coverage), trying a few non–power-of-two factors could fine-tune the optimal pixel size.

(Remarks on code availability)
The ReScale4DL repository provides a Python package and accompanying notebooks for systematically down- and up-sampling annotated light-microscopy images, retraining segmentation models (U-Net, StarDist, and Vision Transformers) at each scale, and measuring IoU to identify an optimal pixel-to-object-diameter ratio (~ 4 %–16 %). Users can install via pip, explore example workflows in

Jupyter notebooks, and adapt the pipeline to their own datasets.

Good Things
• Clear Organization: Code is neatly structured under src/rescale4dl/ with separate notebooks for rescaling, model evaluation, and foundation-model experiments.
• Notebook Examples: Step-by-step notebooks demonstrate how to resample images, train segmentation networks at multiple scales, and compute IoU, making it easy to see end-to-end functionality.
• Installable Package: A pyproject.toml and Makefile allow installation via pip install rescale4dl, facilitating integration into other projects.
• Support for Multiple Architectures: Built-in support for both U-Net/StarDist and pretrained Vision Transformers (MicroSAM) shows versatility across model types.
• Basic Testing Framework: A tests/ folder provides a foundation for quality control, even if coverage is still minimal.

Suggestions to consider
• Centralize Hyperparameters: There's no single file listing learning rates, batch sizes, or epoch counts. Adding a config (e.g., config.yaml or JSON) would help users reproduce exact settings without combing through notebooks.
• Add CLI Entry Points: Currently, all functionality is accessed through notebooks. Providing simple command-line scripts (e.g., rescale4dl rescale … and rescale4dl evaluate …) would enable batch runs and easier integration into automated pipelines.
• Expand Test Coverage: The existing tests/ directory lacks specific unit tests. Including tests that verify image dimensions after rescaling or validate IoU on a toy example would boost confidence.
• Pin Dependencies & Tag Releases: Dependencies (e.g., PyTorch, StarDist versions) aren't explicitly specified in pyproject.toml; adding version bounds and tagging the commit used for the paper would prevent future incompatibilities.
• Document Pretrained Models: The notebook that loads MicroSAM doesn't describe where to download required checkpoints or which version to use. A "Prerequisites" section with links and memory requirements would reduce setup friction.

Reviewer #2

(Remarks to the Author)
In this work by Ferreira et al., the authors present ReScale4DL, a systemic approach for determining the optimal image resolution for the segmentation task when using deep-learning with biological images. More specifically, they link the segmentation accuracy to the receptive field of the networks, and shows that high-resolution acquisitions may be inefficient for segmentation, leveraging on reducing data and computing needs.

Addressing the receptive filed of neural networks as an essential parameter for segmentation is important to shed light on, and this is a noteworthy contribution by the authors. However, the approach remains relatively underdeveloped at this stage. Additional development and analysis are necessary to strengthen the authors' contribution.

First, the bibliography lacks at least two papers that address receptive fields in biomedical image segmentation in a more comprehensive way:
[1] Loos et A. Demystifying the Effect of Receptive Field Size in U-Net Models for Medical Image Segmentation.
[2] Sytwu et al. Understanding the Influence of Receptive Field and Network Complexity in Neural Network-Guided TEM Image Analysis.
Both papers relate the segmentation accuracy to the receptive field and contrast (i.e. SNR), one feature that is completely omitted by the authors. In addition, they also show a more nuanced result concerning the receptive field.

Starting by the end, the authors demonstrate that you can downsample images while maintaining quantification capabilities, having implications for experimental design. I agree with the authors that this can be added to the elements to consider when designing an experiment alongside questions related to the type of microscopy, the labelling, etc.

However, there are also many examples for which you cannot reduce resolution of your experiment, and in this case ReScale4DL does not provide a robust solution. As the authors focus on the receptive field, I would find it very interesting if they push forward this idea with more experiments. For instance, they state: "Conversely, at high resolution, nuclei images can display heterogeneous structures, introducing noise during segmentation and potentially increasing false negatives. This computational perspective contrasts with traditional microscopy practices, where image quality has been equated with higher resolution and magnification, assuming finer details provide more informative content". But, in the next paragraph, they link the accuracy to the receptive field, i.e. context "When image resolution exceeds the network's architectural optimum, pixels lack sufficient contextual information, compromising the model's performance.". These two things different. To really prove that finer details can be detrimental to the segmentation accuracy, they need to define receptive fields in physical units and keep them constant with varying resolutions (i.e. the receptive field in pixel will be bigger for higher resolution), and then compare the segmentation accuracy.

When high resolution is required (for the object texture for instance), one could still downsample the image to perform segmentation. But then, would it be as easy as upsampling the masks to have a proper high-resolution segmentation. Examining Fig4, I would suspect that results could be underwhelming if downsampling was too important. It could be interesting to test until what resizing such an upsampling would be valuable.

Going back to Fig 4, maybe I'm not understanding the plots c) but the text states "Only at coverage ratios exceeding 11% (downsampling factor > 23) did diameter estimates begin to diverge" while I'm feeling like the difference between the GT and prediction is smaller starting from 11%. Why? Fig 4 is also used to demonstrate that phenotypic discrimination is preserved even when downsampling occurs. The size difference between WT and Treatment is quite important (35 pixels versus 50 pixels) hence why the difference is still discriminable. I think the authors should add more conditions with varying radii (by changing the treatment concentration?) to identify the point at which differences become indistinguishable.

In [1], the authors developed a tool to determine the recommend receptive field size with respect to images/masks datasets:
https://github.com/vinloo/u-net-receptive-field-study/tree/master/recommender-tool
How does it relate to your % of coverage factor? What about resizing the pair of images/masks and running the tool. Does it give similar results as ReScale4DL? If yes, it would prevent training the networks.

Finally, I think it's important that authors test their methods with images exhibiting lower SNR, as I'm suspecting that it could impact the downsampling factor.

Minor comments:
- In Fig3-4, it could be interesting to add the receptive field size and the image size (in addition to the % diameter per pixel).
- In Fig 4, I would like to see images for all the conditions, in order to see the segmentation results of the 37% and 44%.
- Why do you use diameter as a quantifier? This seems suboptimal for bacteria that are elongated. In addition, you have access to the masks, so using the area seems more straightforward and accurate.

(Remarks on code availability)

Reviewer #3

(Remarks to the Author)
In this paper, the authors report the effect of rescaling image data on the accuracy of deep-learning-based segmentation. Quantitative comparison of the accuracy clearly shows that there is an optimal level of image rescaling that improves the accuracy. Preprocessing by rescaling image data has been known as a trick among image analysts, but this paper presents concrete evidence of the advantage of this preprocessing step. Overall, the paper meets the general interest of the biological community where bioimage analysis using deep learning based methods is becoming standard, especially since it affects the design of the upstream imaging workflow design as shown in Fig.2.

The paper is associated with sample image data, reproducible processing codes to systematically rescale image data, parameters required for deep-learning-based segmentations, and reproducible analysis codes for plotting the accuracies with different levels of rescaling. With modifications and corrections of code in the GitHub repository explained below, I recommend this paper to be published in Nature Communications.

Overall Comments

The paper shows that rescaling the original image to a smaller image increases the accuracy of image segmentation using a CNN-based algorithm. This is an advantage for measuring morphological parameters of the segmented region. However, the delineated region by such a segmentation step is often used for measuring other features, such as intensities and object distribution within that region in a different channel. It would be better to have some explanation of the applicability of the rescaling step in such workflows. Would it be better to avoid rescaling in such a multi-channel measurement case, or does the advantage of increased segmentation accuracy exceed the disadvantage of the loss of resolution in other channels?

Another idea, instead of rescaling, is to adjust the size of the reception field in the CNN. This might be naturally avoided because the effect is too much, but it can be discussed why this was not an option.

Throughout the paper, a measure called "% diameter per pixel" is used to show the extent of rescaling. As the quality of the segmentation results relies on the relative size of objects, this may be a good indicator to gauge the expected accuracy of segmentation results. However, the use of this measure in the results was confusing to me. It would be better if the rescaling value were directly shown instead of % diameter per pixel, because these percentage values change depending on the size of the segmentation target, even when the rescaling value is identical. Especially when two different populations with differences in diameter are compared, such as the case in Fig. 4, this confusion is maximized. In Fig. 4a, wild type 22.4% diameter per pixel and treated 19% diameter per pixel probably are with the same rescaling level, but as the corresponding % diameter per pixel values become different, it was confusing and made me wonder why these two seemingly different conditions can be compared. In Fig. 4c and d, the horizontal axis is % diameter per pixel for showing the difference between the two populations; only the % diameter per pixel of one of the populations is shown in the axis label. In the GitHub Jupyter notebook Evaluate_Segmentation.ipynb, the other population is shown - I think the authors are slightly confused as well. To overcome such confusions, it is better to directly show the rescaling values (2, 2^2, 2^3 ...) in these figures, and add a new table to list these rescaling values and % diameter per pixel to show their relationship. For example:

Rescaling | % pixel diameter WT | % pixel diameter Treatment

2^0 | 2.8 | 2.4

2^1 | 5.7 | 4.7

2^2 | 11.4 | 9.5

2^3 | 22.9 | 19.0

Minor Comments

page 1 Fig.1c: In this figure, there are three circular gray icons: 1) with two small white circles, 2) flat gray, and 3) with a broad black rim. There is no explanation about these icons, but it seems that these icons each represent 1) "fragmented segmentations caused by non-continuous edges and inner pixels misclassified as background, referred to as false negatives", 2) almost perfect segmentation, and 3) "over-generalization at edges". I think these icons and these explanations are better to be explicitly linked, as the same icons are also used in Figs 3a and c.

page 3 Fig 3b, d: What do the white circles in the plots represent?

page 3 Fig 3 legend "binary mask (merged label)": It took me some while to figure out that this means "merged = edge + inner region". Maybe add a bit more like: "binary mask (merged label = edge + inner region)"?

Page 4 column 1, 2nd paragraph: missing Note number and Figure number

Page 4 column 2, "segmentation networks" and "Evaluation": missing Table number

Page 4 column 2, "the diameter Dij of non-rounded shapes (C. elegans and E. coli datasets) was computed as the median of the Euclidean Distance Transform (EDT) values contained in the object's skeleton. " It would be great to have the script used for this measurement in GitHub, and references, if existing, that use this seemingly new method for determining the diameter of elongated shapes.

There is a bug in the GitHub repo, Rescale_Images.ipynb. Line 124: all parameters should be capitalized to use the values defined in Line82-86.
(https://github.com/HenriquesLab/ReScale4DL/blob/dc18418a61de9072bfb22ad18674195ff8e1cf8d/Notebooks/Rescale_Images.ipynb#L124)

Supplementary Figure 5

The results shown in this figure are not associated with method details, and the explanation and interpretation of these results are immature. I could not understand how this was done or where I should focus. My impression is that this figure should be in a different new paper. Otherwise, it should be a part of the main text, not as a supplementary, as this figure itself seems to be a new finding, not appropriate for being "supplementary" to what is shown in the main text.

(Remarks on code availability)
My comments on the code published in the GitHub repository are within the "Comments for the Author" written above.

Version 1:

Reviewer comments:

Reviewer #2

(Remarks to the Author)
The revised version of the manuscript is greatly improved. The authors have addressed all my suggestions and concerns effectively. I recommend its publication.

(Remarks on code availability)
README is present and has enough information to install and run the code.

Reviewer #3

(Remarks to the Author)
I thank the authors for responding to my comments and reflecting my suggestions in the revised manuscript. The manuscript is ready for publication.

Aside from the manuscript itself, the code in GitHub does not seem to be working well. I added details of my experiences with three Jupyter notebooks listed in the README in below. The publication will be fully ready when these issues are solved. Asking a third person to test notebooks without giving any instructions might help fix these problems.

1. Rescale_Images.ipynb : This notebook is OK.
2. Evaluate_Segmentation2D.ipynb (The top README needs to be updated):

with INPUT_DIR = "Examples/segmentation_results/Saureus_instance_segmentation"
The 2nd cell returned following error

```
Calculating properties for OG
Sampling folders to analyze: ['GT', 'Prediction', 'Results']
Done.
No semantic segmentation images found.
Calculating properties for downsampling_4
Sampling folders to analyze: ['GT', 'Prediction', 'Results']
Done.
No semantic segmentation images found.
Calculating properties for downsampling_2
Sampling folders to analyze: ['GT', 'Prediction', 'Results']
Done.
No semantic segmentation images found.
Total time: 00:00:00
```

3. Examples/data2rescale/Saureus_instance_segmentation/Images
The 3rd cell returned the following errors

```
Rescaling and cropping: 0%| | 0/4 [00:00<?, ?it/s]
10.0
---------------------------------------------------------------------------
OverflowError Traceback (most recent call last)
Cell In[3], line 1
----> 1 rescale_and_crop(INPUT_DIR, OUTPUT_DIR, SCALE_FACTOR, TARGET_SHAPE, SAVE_SCALED)
2 print(f"Processing complete! Results saved to: {OUTPUT_DIR}")
```

File ~/.cache/uv/archive-v0/P6KrUh-emdBSv6Zd/lib/python3.12/site-packages/rescale4dl/batch.py:209, in rescale_and_crop(INPUT_DIR, OUTPUT_DIR, SCALE_FACTOR, TARGET_SHAPE, SAVE_SCALED)
207 SCALE_MODE = "up"
208 print(s)
--> 209 rescale_and_crop_image(INPUT_DIR, OUTPUT_DIR, np.int8(s), SCALE_MODE, TARGET_SHAPE, SAVE_SCALED)

File ~/.cache/uv/archive-v0/P6KrUh-emdBSv6Zd/lib/python3.12/site-packages/rescale4dl/batch.py:187, in rescale_and_crop_image(INPUT_DIR, OUTPUT_DIR, SCALE_FACTOR, SCALE_MODE, TARGET_SHAPE, SAVE_SCALED)
184 image = np.squeeze(imread(img_path))
186 # Rescale
--> 187 scaled = rescale_image(image, SCALE_FACTOR, SCALE_MODE)
189 # Save intermediate
190 if SAVE_SCALED:

File ~/.cache/uv/archive-v0/P6KrUh-emdBSv6Zd/lib/python3.12/site-packages/rescale4dl/batch.py:166, in rescale_image(image, factor, mode)
164 """Rescale image using specified method"""
165 if mode == "down":
--> 166 return binning_img(image, factor, keep_dims=False, mode="mean")
167 elif mode == "up":
168 return upsample_img(image, factor, keep_dims=False)

File ~/.cache/uv/archive-v0/P6KrUh-emdBSv6Zd/lib/python3.12/site-packages/rescale4dl/downscaling.py:61, in binning_img(img, downsampling_factor, keep_dims, mode)
59 return binning_blur(img, downsampling_factor, mode=mode)
60 else:
---> 61 return binning_downsize(img, downsampling_factor, mode=mode)

File ~/.cache/uv/archive-v0/P6KrUh-emdBSv6Zd/lib/python3.12/site-packages/rescale4dl/downscaling.py:16, in binning_downsize(img, downsampling_factor, mode)
9 def binning_downsize(img: np.array, downsampling_factor: int, mode: str = "sum"):
10 """Bins a 2D array by a given factor. The last two dimensions of the array are binned.
11 :param arr: numpy array with any shape as long as last two dimensions are y, x (example: time, channel, z, y, x)
12 :param bin_factor: factor used to bin dimensions
13 :param mode: can be either sum or mean, defaults to sum if not specified or not valid mode
14 :return: binned array
15 """
---> 16 return rebin_2d(img, downsampling_factor, mode=mode)

File ~/.cache/uv/archive-v0/P6KrUh-emdBSv6Zd/lib/python3.12/site-packages/nanopyx/core/transform/binning.py:21, in rebin_2d(arr, bin_factor, mode)
18 if bin_factor < 1:
19 raise ValueError("Binning factor must be greater than 1")
---> 21 if arr.shape[-1] % bin_factor != 0 or arr.shape[-2] % bin_factor != 0:
22 raise ValueError("Binning factor must be a divisor of the last two dimensions")
24 if mode == "sum":
OverflowError: Python integer 512 out of bounds for int8

(Remarks on code availability)
1. Rescale_Images.ipynb : This notebook is OK.

2. Evaluate_Segmentation2D.ipynb (The top README needs to be updated with the new file name):

with INPUT_DIR = "Examples/segmentation_results/Saureus_instance_segmentation"
The 2nd cell returned following error

Calculating properties for OG
Sampling folders to analyze: ['GT', 'Prediction', 'Results']
Done.
No semantic segmentation images found.
Calculating properties for downsampling_4
Sampling folders to analyze: ['GT', 'Prediction', 'Results']
Done.
No semantic segmentation images found.
Calculating properties for downsampling_2
Sampling folders to analyze: ['GT', 'Prediction', 'Results']
Done.
No semantic segmentation images found.
Total time: 00:00:00

3. Examples/data2rescale/Saureus_instance_segmentation/Images
The 3rd cell returned the following errors
Rescaling and cropping: 0%| | 0/4 [00:00<?, ?it/s]
10.0

OverflowError Traceback (most recent call last)

```
Cell In[3], line 1
----> 1 rescale_and_crop(INPUT_DIR, OUTPUT_DIR, SCALE_FACTOR, TARGET_SHAPE, SAVE_SCALED)
2 print(f"Processing complete! Results saved to: {OUTPUT_DIR}")

File ~/.cache/uv/archive-v0/P6KrUh-emdBSv6Zd/lib/python3.12/site-packages/rescale4dl/batch.py:209, in rescale_and_crop(INPUT_DIR, OUTPUT_DIR, SCALE_FACTOR, TARGET_SHAPE, SAVE_SCALED)
207 SCALE_MODE = "up"
208 print(s)
--> 209 rescale_and_crop_image(INPUT_DIR, OUTPUT_DIR, np.int8(s), SCALE_MODE, TARGET_SHAPE, SAVE_SCALED)

File ~/.cache/uv/archive-v0/P6KrUh-emdBSv6Zd/lib/python3.12/site-packages/rescale4dl/batch.py:187, in rescale_and_crop_image(INPUT_DIR, OUTPUT_DIR, SCALE_FACTOR, SCALE_MODE, TARGET_SHAPE, SAVE_SCALED)
184 image = np.squeeze(imread(img_path))
186 # Rescale
--> 187 scaled = rescale_image(image, SCALE_FACTOR, SCALE_MODE)
189 # Save intermediate
190 if SAVE_SCALED:

File ~/.cache/uv/archive-v0/P6KrUh-emdBSv6Zd/lib/python3.12/site-packages/rescale4dl/batch.py:166, in rescale_image(image, factor, mode)
164 """Rescale image using specified method"""
165 if mode == "down":
--> 166 return binning_img(image, factor, keep_dims=False, mode="mean")
167 elif mode == "up":
168 return upsample_img(image, factor, keep_dims=False)

File ~/.cache/uv/archive-v0/P6KrUh-emdBSv6Zd/lib/python3.12/site-packages/rescale4dl/downscaling.py:61, in binning_img(img, downsampling_factor, keep_dims, mode)
59 return binning_blur(img, downsampling_factor, mode=mode)
60 else:
---> 61 return binning_downsize(img, downsampling_factor, mode=mode)

File ~/.cache/uv/archive-v0/P6KrUh-emdBSv6Zd/lib/python3.12/site-packages/rescale4dl/downscaling.py:16, in binning_downsize(img, downsampling_factor, mode)
9 def binning_downsize(img: np.array, downsampling_factor: int, mode: str = "sum"):
10 """Bins a 2D array by a given factor. The last two dimensions of the array are binned.
11 :param arr: numpy array with any shape as long as last two dimensions are y, x (example: time, channel, z, y, x)
12 :param bin_factor: factor used to bin dimensions
13 :param mode: can be either sum or mean, defaults to sum if not specified or not valid mode
14 :return: binned array
15 """
---> 16 return rebin_2d(img, downsampling_factor, mode=mode)

File ~/.cache/uv/archive-v0/P6KrUh-emdBSv6Zd/lib/python3.12/site-packages/nanopyx/core/transform/binning.py:21, in rebin_2d(arr, bin_factor, mode)
18 if bin_factor < 1:
19 raise ValueError("Binning factor must be greater than 1")
---> 21 if arr.shape[-1] % bin_factor != 0 or arr.shape[-2] % bin_factor != 0:
22 raise ValueError("Binning factor must be a divisor of the last two dimensions")
24 if mode == "sum":
OverflowError: Python integer 512 out of bounds for int8
```

Version 2:

Reviewer comments:

Reviewer #3

(Remarks to the Author)
I tested the updated notebooks. They are now running without any errors, ready for publication.
I thank the authors for their effort to fix these errors.

(Remarks on code availability)
Notebooks are now without any errors.

Open Access This Peer Review File is licensed under a Creative Commons Attribution 4.0 International License, which permits use, sharing, adaptation, distribution and reproduction in any medium or format, as long as you give appropriate credit to the original author(s) and the source, provide a link to the Creative Commons license, and indicate if changes were made.
In cases where reviewers are anonymous, credit should be given to 'Anonymous Referee' and the source.
The images or other third party material in this Peer Review File are included in the article's Creative Commons license, unless indicated otherwise in a credit line to the material. If material is not included in the article's Creative Commons license and your intended use is not permitted by statutory regulation or exceeds the permitted use, you will need to obtain permission directly from the copyright holder.
To view a copy of this license, visit https://creativecommons.org/licenses/by/4.0/

REVIEWER COMMENTS

Reviewer #1 (Remarks to the Author):

Short Summary
Ferreira et al. introduce ReScale4DL, a pipeline that systematically resamples annotated light-microscopy images at multiple scales and retrains segmentation models (U-Net, StarDist) on each version. By measuring Intersection-over-Union (IoU), they pinpoint an optimal pixel-to-object-diameter ratio (~ 4 %–16 %) that maximizes accuracy while dramatically boosting throughput. Tested on 2D brightfield, phase-contrast, and SIM datasets, imaging at this "sweet spot" yields smoother masks and enables 15x–40x faster data collection without sacrificing performance. The key insight is that highest resolution isn't always best—matching pixel size to the model's effective receptive field (~ 4 %–16 % of object diameter) optimizes both segmentation and experimental efficiency.

Recommendation

This reviewer finds the study suitable for publication. Consideration of the feedback below would strengthen the study.

Major Feedback

R1.1 It would be helpful to include key training hyperparameters (learning rate, batch size, number of epochs). This reviewer looked through the paper and the GitHub repo but couldn't readily find them, and the referenced table ("Table ??") in the GitHub repo readme appears missing.

Our reply: We apologise for the confusion in referencing the table of hyperparameters in the Supplementary Material. We have improved the information in the text with a more explicit reference:

> (<u>Methods section</u>) *"Segmentation networks. The 2D Multilabel U-Net and 2D StarDist (9) models were trained using the respective ZeroCostDL4Mic (2) notebooks in local GPU-equipped workstations with DL4MicEverywhere (11) and the hyperparameter configuration specified as in Table 1 in Supplementary Note 1."*
>
> (<u>Methods section</u>) *"Evaluation. The Intersection over Union (IoU) scores visualised in Figs. 3 and 4 were calculated for the results of all the models specified in Table 1 in Supplementary Note 1, and ..."*

After thorough inspection, we did not find any other reference to the Table 1 of hyperparameters in the GitHub repository of ReScale4DL. Nevertheless, we have extended the documentation in the repository to point users to the tools we used to train the proposed deep learning networks as:

> **Additional DL resources for microscopy:**
> The deep learning networks presented in the ReScale4DL paper were trained using the following platforms:
>
> - **ZeroCostDL4Mic**: A Google Colab-based no-cost toolbox to explore Deep Learning in Microscopy
> - **DL4MicEverywhere**: Docker-based implementation bringing the ZeroCostDL4Mic experience for local deployment
>
> For detailed hyperparameter settings and training configurations, please refer to **Table 1** in our bioRxiv preprint.

R1.2 Similarly, adding sample sizes and a simple statistical test to the IoU results would make it clearer how robust the differences are. I couldn't find that information in the paper or repo.

Our reply: We have integrated this information in the Supplementary Note 4 in a new figure:

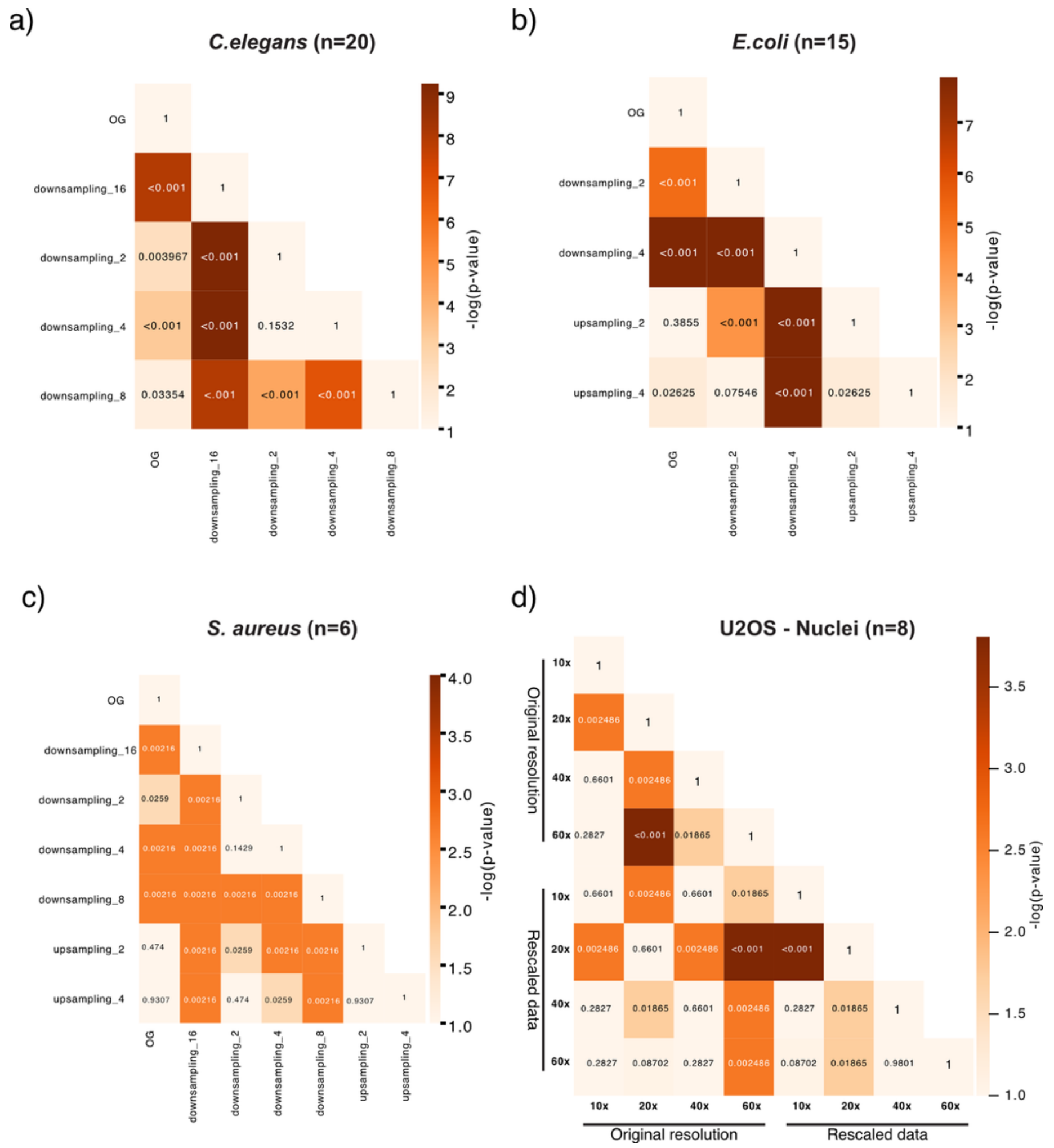

Fig. 7. Kolmogorov-Smirnov statistical hypothesis tests. *IoU mean value distribution for (a-b) the semantic segmentation of C. elegans (Fig. 3e) and E. coli (Fig. 3b), and (c-d) the instance segmentation of S. aureus (Fig. 5b) and U2OS nuclei (Fig. 4b) were compared using the Kolmogorov-Smirnov statistical hypothesis test. For each image an IoU value is obtained and the n value corresponds to the total number of images. Being an instance segmentation task, in c) and d), the IoU of each cell in the image is calculated and the average IoU of all the cells in the image is the one considered for the statistical test.*

Additionally, all the notebooks contain now an additional section (*Statistical Analysis*) to compute a series of statistical hypothesis tests, either automatically based on the data distribution, or one between t-test, Kolmogorov-Smirnov or Welch's t-test. This section will also inform about the sample size and statistical significance.

R1.3 All experiments use 2D light-microscopy images. Testing on a 3D EM dataset (e.g., FIB-SEM) would show whether the 4 %–16 % pixel-diameter guideline holds for volumetric data. Because all tests are on light microscopy, including an EM example would confirm that the "sweet spot" generalizes to higher-resolution modalities.

Our reply: Following the reviewer's suggestion we assessed the performance of the 3D U-Net to segment mitochondria in EM images taken from the CA1 hippocampus region of the brain

(https://www.epfl.ch/labs/cvlab/data/data-em/). These image volumes have an isotropic resolution of 5x5x5nm. We tested the effect of varying resolutions in the results of a 3D U-Net considering lateral, axial and lateral & axial rescaling. These new experiments are included in an updated version of the Figure 3 (shown below). Importantly, in 3D microscopy imaging there is often more flexibility in choosing alternative lateral (XY) resolutions than axial (Z). The resolution of 3D volumes is unlikely isometric and tends to be lower in Z than in XY. Therefore, it is less frequent to be in the situation of having an excessive axial resolution.

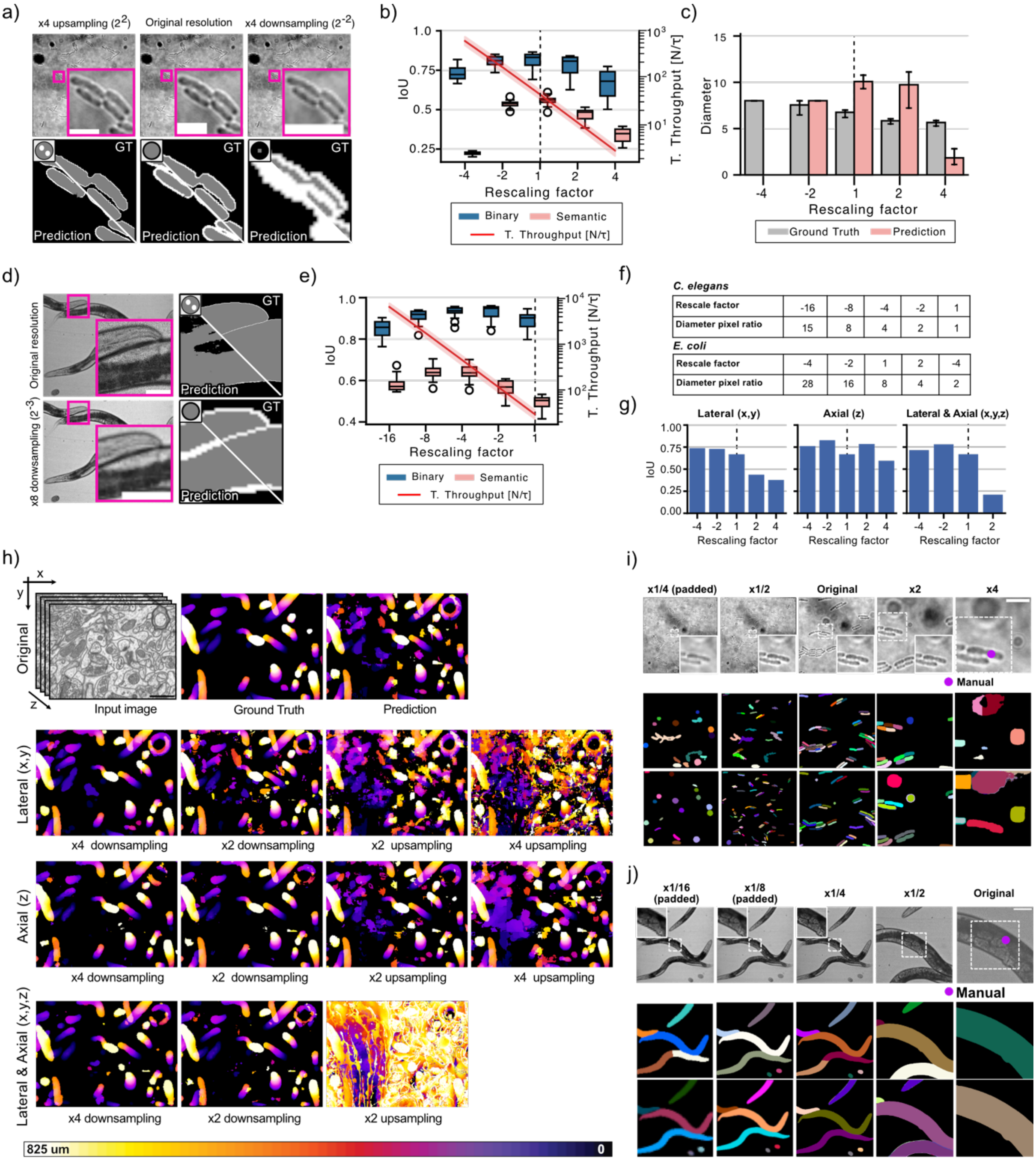

Fig. 3. Impact of image resolution on deep learning segmentation accuracy. a)-f) Results of 2D U-Net models trained for semantic segmentation (inner area and boundary) of *E. coli* a) and *C. elegans* d) at varying simulated resolutions. b) Intersection over Union (IoU) distributions (semantic and binary masks) across scaling factors for *E. coli*, ranging from $\times -4$ to $\times 4$. IoU was calculated both for semantic segmentation (average IoU of edge and inner regions) and the binary masks resulting from considering the edge and inner regions. Purple line (log scale): theoretical throughput calculated as the number of objects observable by a unit of time (τ) given a certain resolution. c) *E. coli* diameter distributions from U-Net segmentations versus

ground truth. e) Same as in b), IoU for *C. elegans* segmentation at varying resolutions. g) IoU on the test image volume of 3D U-Net mitochondria binary segmentation in transmission electron microscopy (TEM) at varying resolutions. h) Results visualizations corresponding to z-colour-coded-projections at varying axial/lateral resolutions. i-j) MicroSAM (1) with a single point-wise prompting strategy was used to obtain the instance segmentation of *E. coli* (i) and *C. elegans* (j) example images. We pointed to the same object in each rescaled image and run the automatic instance segmentation pipeline. All the images matched model input dimensions (512 × 512 pixels) to avoid internal tiling or image re-scaling. The images that had smaller dimensions were padded with zeros. Square boxes correspond to the same physical area on each image. Scale bars correspond to a) 100, 25, and 10 pixels; d) 100 and 25 pixels; h) 200 pixels; i)-j) 50 µm. Circles in b) and e) correspond to data points outside the quartiles of the IoU distribution.

Minor Feedback

R1.4: Terminology around "image resolution" vs. "pixel size" is used inconsistently; a brief early definition distinguishing these terms would improve clarity.

Our reply: As suggested by the reviewer, these terms are further defined in the text together with a clearer example of what terms we are referring to. The information is updated in the introduction as:

> *"For clarity, we define image resolution as the information content per pixel, separate from optical resolution considerations and directly relatable to pixel sizes, defined as the physical measurement of one pixel. For instance, cell nuclei appear more homogeneous at lower resolutions, enabling neural networks to generalise more effectively. Conversely, at high resolution, nuclei images, which usually have a higher number of pixels to analyse, can display heterogeneous structures, introducing noise during segmentation and potentially increasing false negatives."*

R1.5: In the Discussion (right after mentioning MicroSAM, reference 23), there are placeholders "Note ??, Fig. ??" that need updating before publication.

Our reply: We have now corrected in the main text as

> *"As demonstrated through MicroSAM (1) foundation model evaluation, fixed context windows in pre-trained models remain susceptible to resolution mismatches (Fig. 3(i-j) and Supp. Fig. 7).*

R1.6: The throughput curves (Figures 3b, 3d, and 4b) are plotted on a logarithmic axis but do not include clear axis labels or units (e.g., "objects per second"), which could confuse readers.

Our reply: The estimation of throughput is given in the Methods section (Equation 3) as an estimate of the number of objects that can be captured by a unit of time, being N the number of objects (e.g., cells) and the Latin "tau" (τ), a unit of time. These units are indicated in the graphs corresponding to Figure 3b, 3d and 4b, in the right Y-axis as T. Throughput [N/ τ]. To further ease the visual inspection, we have included this information in the plot legends.

R1.7: Some legends use both "x" notation (e.g., "2x downsampling") and percentage coverage interchangeably; standardizing to one style would be preferable.

Our reply: We have updated the information regarding image rescaling. For clarity, all figures now include the scaling factor and we have integrated three tables with the relationship between the image rescaling and the percentage of an object diameter covered by a pixel (Fig. 3f (shown above) and Table 3 in the Supplementary Note 3 (shown below)).

Table 2. Rescaling factors for *S. aureus*. The rescaling factor indicates data upsampling or downsampling, depending on whether it is higher or lower than one, respectively. Pixel diameter [%] refers to the proportion of the diameter covered by one pixel for the wild type and treated bacteria

samples.

S. aureus	Pixel Diameter [%]	
**Rescaling factor**	**Wildtype**	**Treatment**
2^2	0.71	0.5
2	1.41	1.0
**1**	**2.84**	**2.1**
2^{-1}	5,67	4.1
2^{-2}	11,35	8.2
2^{-3}	22,73	16.5
2^{-4}	45,20	32.8

R1.8: Color choices in plots (especially the purple throughput curve) may be hard to distinguish in grayscale or for color-blind readers; adding dashed lines or markers would help.

Our reply: We thank the reviewer for the suggestion. The throughput line is now included in basic red and it is thickened (see the Fig. 3 above). It is the only diagonal curve in the plot, which we believe avoids further confusion.

R1.9: Small typos and formatting inconsistencies appear (e.g., extra line breaks splitting sentences in the Methods); a final proofread would catch these.

Our reply: We thank the reviewer for highlighting this aspect. We have thoroughly revised the manuscript, and its format and wording should now be adequate.

R1.10: While code availability is noted, version tags or commit hashes for the ReScale4DL repository are missing, which would aid reproducibility.

Our reply: This information is now explicit in the manuscript. Please, note that upon community feedback and further reviews, this could potentially change:

> "The code for image rescaling and metric computation is available at https://github.com/HenriquesLab/ReScale4DL under version ReScale4DL - 0.2.2."

Of Interest, but Not Essential

R1.11: The throughput gains are modeled, but adding a brief real-world example (e.g., a live-cell time course) would illustrate how downsampling impacts photobleaching or frame rates in practice.

Our reply: We thank the reviewer for this suggestion. The relationship between optical resolution and photobleaching is not straightforward: in widefield imaging, higher-NA objectives collect more photons, which typically allows laser power to be reduced and partially offsets the photodamage associated with higher-resolution acquisition. The clearer practical benefit lies elsewhere: lower-resolution acquisition is markedly faster, and when a fixed FOV must be tiled at high magnification, the tile overlaps receive cumulative exposure and accumulate photodamage. To quantify both effects, we added new experimental data on U2OS cell nuclei stained with Hoechst, acquired at 10x, 20x, 40x and 60x, from which we measured the per-FOV acquisition time and the number of tiles required at each magnification. The corresponding additions to the manuscript read as follows:

> (Results section, Optimising image resolution during acquisition improves segmentation accuracy and throughput) *Remarkably, 2D acquisition of the same FOV, considering an average displacement through the wheel, took 3.91, 9.51, 17.12, 36.48 seconds at 10×, 20×, 40× and 60×, respectively, implying that the acquisition at optimal quantitative conditions (20×) is also*

~4× faster than at 60×. Notwithstanding, the FOV at 20× required a 3×3 tiling, while for 60× a tiling of 7×7 was needed, significantly increasing the exposure to fluorescence and therefore, the induced photodamage, in the overlapping regions.

(Discussion) *These findings together have profound implications for experimental design: researchers can substantially increase imaging throughput, speed up image acquisition, increase temporal resolution, reduce indirect photobleaching and phototoxicity resulting from tiling, minimise data storage requirements, and accelerate analysis pipelines—all without compromising biological insights (23, 24).*

R1.12: Since downsampling only by powers of two might skip "in-between" scales (like ~10 % coverage), trying a few non–power-of-two factors could fine-tune the optimal pixel size.

Our reply: The new U2OS cell nuclei dataset addresses this directly, since it spans non-power-of-two scales from real acquisition setups: the scaling factors are x2 (20x), x4 (40x) and x6 (60x) relative to the 10x acquisition. More generally, ReScale4DL accepts arbitrary user-specified scaling factors and is not restricted to powers of two. We deliberately frame the analysis around the acquisition step, where the objective choice typically fixes the pixel size, so the most informative factors to test are those that map onto magnifications a user can realistically achieve at the microscope.

Reviewer #1 (Remarks on code availability):

The ReScale4DL repository provides a Python package and accompanying notebooks for systematically down- and up-sampling annotated light-microscopy images, retraining segmentation models (U-Net, StarDist, and Vision Transformers) at each scale, and measuring IoU to identify an optimal pixel-to-object-diameter ratio (~ 4 %–16 %). Users can install via pip, explore example workflows in Jupyter notebooks, and adapt the pipeline to their own datasets.

Good Things

• Clear Organization: Code is neatly structured under src/rescale4dl/ with separate notebooks for rescaling, model evaluation, and foundation-model experiments.

• Notebook Examples: Step-by-step notebooks demonstrate how to resample images, train segmentation networks at multiple scales, and compute IoU, making it easy to see end-to-end functionality.

• Installable Package: A pyproject.toml and Makefile allow installation via pip install rescale4dl, facilitating integration into other projects.

• Support for Multiple Architectures: Built-in support for both U-Net/StarDist and pretrained Vision Transformers (MicroSAM) shows versatility across model types.

• Basic Testing Framework: A tests/ folder provides a foundation for quality control, even if coverage is still minimal.

Suggestions to consider

R1.13: Centralize Hyperparameters: There's no single file listing learning rates, batch sizes, or epoch counts. Adding a config (e.g., config.yaml or JSON) would help users reproduce exact settings without combing through notebooks

Our reply: All this information is in the Table 1 from the Supplementary Material, directly linking the parameters to the results presented in the manuscript. Additionally, we have made all the trained models publicly available (https://zenodo.org/records/20040567). Each of these models has an additional PDF file exported from the notebook by the time of training the network, indicating the specific hyperparameters used to train each of the models. This information is available in the Data availability statement as follows:

(Data availability section) All the trained models are also available in Zenodo (https://zenodo.org/records/20040567). These models can all be loaded, tested and retrained through the respective notebooks in the DL4MicEverywhere platform (12).

R1.14: Add CLI Entry Points: Currently, all functionality is accessed through notebooks. Providing simple command-line scripts (e.g., rescale4dl rescale … and rescale4dl evaluate …) would enable batch runs and easier integration into automated pipelines.

Our reply: We thank the reviewer for the suggestion. The ReScale4DL package includes basic Python API functionalities that could be quickly run in a very short script. These are now described in the documentation in GitHub as follows:

ReScale4DL provides a set of functionalities to quickly analyse your images and find an optimal pixel size:

1. Rescaling the images in the path by donwsampling with a factor of 2 and 3, and by upsampling with a factor of 2:

   rescale4dl.batch.process_all_datasets("/path/data", [2,3], [2], [1], modes=["mean"])

2. Analyse the segmentation results for different scaling factors in 2D:

   rescale4dl.analyse("/path/data")

3. Analyse the segmentation results for different scaling factors in 3D:

```
rescale4dl.analyse("/path/data",
                   is_3d=True,
                   run_per_object_stats = False, # True for Instance Segmentation, False for Semantic or
Binary Segmentation
                   save_images = False, # True to save images of the segmentation examples and data
distributions, False to skip saving images and saving some memory
                   sampling_dir_list = None)
```

We consider that researchers interacting with the package through a CLI, would already be advanced enough also to also quickly run these lines in the terminal or any other development environment.

R1.15: Expand Test Coverage: The existing tests/ directory lacks specific unit tests. Including tests that verify image dimensions after rescaling or validate IoU on a toy example would boost confidence.

Our reply: We included automated testing for the package in the GitHub repository to ensure the code runs correctly. These can be found at https://github.com/HenriquesLab/ReScale4DL/tree/main/tests.

R1.16: Pin Dependencies & Tag Releases: Dependencies (e.g., PyTorch, StarDist versions) aren't explicitly specified in pyproject.toml; adding version bounds and tagging the commit used for the paper would prevent future incompatibilities.

Our reply: All the dependencies needed to reproduce the code in ReScale4DL are explicitly described in the project.toml file, including dependencies bounds. ReScale4DL does not use any deep learning specific python package (e.g., TensorFlow, PyTorch, StarDist) nor load any deep learning model, as the code is not meant to train or deploy deep learning models. The models presented in the paper were trained, evaluated and used with DL4MicEverywhere and MicroSAM. These two software tools are published, publicly available and their environment is explicitly specified in their corresponding installation guidelines. Moreover, all the models trained for the results presented in the manuscript can be deployed in Docker containers with all the specific environment setup already through a tagged

Docker image existing within the DL4MicEverywhere platform. MicroSAM was used via its Napari plugin.

R1.17: Document Pretrained Models: The notebook that loads MicroSAM doesn't describe where to download required checkpoints or which version to use. A "Prerequisites" section with links and memory requirements would reduce setup friction.

Our reply: In ReScale4DL there is no notebook loading any deep learning model, including MicroSAM. The only notebook related to foundation models (Rescale_Foundation_Models.ipynb) is meant to only rescale input images in the correct format to then be used with a dedicated software tool. A pretrained model from MicroSAM's collection of models was loaded using MicroSAM's napari plugin. This information is described in the Supplementary Material, Supplementary Note 2 as:

> "*We performed instance segmentation of Escherichia coli (E. coli), Caenorhabditis elegans (C. elegans), and Staphylococcus aureus (S. aureus) samples using MicroSAM (23) software, with the same rescaling factors used for the Figs. 2 and 4. For simplicity, we used their 2D Annotator plugin in Napari to deploy interactive segmentation with a single-point prompt-based approach, followed by the automatic instance segmentation. Rescaled images were individually segmented by manually pointing to the same structural landmark each time. For all experiments, we chose the Vision Transformer (ViT) Large backbone architecture, a fine-tuned version of the original Segment Anything Model (SAM) (26) for cellular and nuclear segmentation in light microscopy (ViT-l-lm).*"

Reviewer #2 (Remarks to the Author):

In this work by Ferreira et al., the authors present ReScale4DL, a systemic approach for determining the optimal image resolution for the segmentation task when using deep-learning with biological images. More specifically, they link the segmentation accuracy to the receptive field of the networks, and shows that high-resolution acquisitions may be inefficient for segmentation, leveraging on reducing data and computing needs.

Addressing the receptive filed of neural networks as an essential parameter for segmentation is important to shed light on, and this is a noteworthy contribution by the authors. However, the approach remains relatively underdeveloped at this stage. Additional development and analysis are necessary to strengthen the authors' contribution.

R2.1: First, the bibliography lacks at least two papers that address receptive fields in biomedical image segmentation in a more comprehensive way:
[1] Loos et A. Demystifying the Effect of Receptive Field Size in U-Net Models for Medical Image Segmentation.
[2] Sytwu et al. Understanding the Influence of Receptive Field and Network Complexity in Neural Network-Guided TEM Image Analysis.
Both papers relate the segmentation accuracy to the receptive field and contrast (i.e. SNR), one feature that is completely omitted by the authors. In addition, they also show a more nuanced result concerning the receptive field.

Our reply: We updated the introduction with the reviewer's feedback which reads as follow (17 and 18 being the references suggested):

> *The limitation stems from convolutional neural network's (CNN) receptive fields—the spatial region influencing a single output pixel. When image resolution exceeds the network's*

architectural optimum, pixels lack sufficient contextual information, compromising the model's performance. Practitioners often address this by downsampling the images during pre-processing. Others, have already related the segmentation accuracy to the receptive field and contrast (i.e., SNR) in medical images (17) and electron microscopy images (18), and propose adapting the networks' architecture to the data features accordingly (17). Incorporating resolution and imaging considerations directly into the experimental design would enable acquisition at optimal resolutions from the outset, simultaneously increasing throughput and reducing computational demands.

R2.1: Starting by the end, the authors demonstrate that you can downsample images while maintaining quantification capabilities, having implications for experimental design. I agree with the authors that this can be added to the elements to consider when designing an experiment alongside questions related to the type of microscopy, the labelling, etc.
However, there are also many examples for which you cannot reduce resolution of your experiment, and in this case ReScale4DL does not provide a robust solution. As the authors focus on the receptive field, I would find it very interesting if they push forward this idea with more experiments. For instance, they state: "Conversely, at high resolution, nuclei images can display heterogeneous structures, introducing noise during segmentation and potentially increasing false negatives. This computational perspective contrasts with traditional microscopy practices, where image quality has been equated with higher resolution and magnification, assuming finer details provide more informative content".
But, in the next paragraph, they link the accuracy to the receptive field, i.e. context "When image resolution exceeds the network's architectural optimum, pixels lack sufficient contextual information, compromising the model's performance.". These two things different. To really prove that finer details can be detrimental to the segmentation accuracy, they need to define receptive fields in physical units and keep them constant with varying resolutions (i.e. the receptive field in pixel will be bigger for higher resolution), and then compare the segmentation accuracy.

Our reply: We thank the reviewer for these important highlights. We agree that our original wording conflated two different effects: the presence of fine structural details and the limitation imposed by insufficient contextual information due to the network receptive field. We have corrected the text accordingly to clarify that the main factor underlying the observed performance degradation at high resolution is the lack of sufficient context available to the network, rather than fine details being inherently detrimental. This reads now as follows:

For instance, while cell nuclei display heterogeneous structures at high resolutions, they are often more accurately segmented at lower resolutions, where sufficient contextual information can be captured using fewer pixels. This computational perspective contrasts with traditional microscopy practices, where image quality has been equated with higher resolution and magnification, assuming finer details provide more informative content (2). However, while visually appealing, the wealth of information in high-resolution images can be irrelevant or even detrimental to computational analysis, as our results here demonstrate.

As suggested by the reviewer and in line with the work referenced in [1], we performed an additional benchmark adapting the receptive field to maintain constant physical units and tested its equivalence to the proposed rescaling approach. We used the *C. elegans* dataset and starting with a baseline U-Net with two pooling operations, we increased the effective receptive field by modifying only the convolutional kernel size and the dilation/stride parameters, thereby approximating the receptive field obtained when downsampling the input images. The obtained results are in line with the ones in [1,2]: increasing the receptive field can recover segmentation performance comparable to image downsampling, and in some cases even yields slightly improved accuracy. These are now included in

the revised version of the manuscript as follows and in Table 1 and Fig. 6 in the supplementary material (below):

> *Objects with diameters exceeding 25 pixels (corresponding to < 4% diameter coverage per pixel) become problematic as their size exceeds the receptive field, while objects smaller than 6 pixels (> 16% diameter coverage) risk information dilution and loss through the network's operations. Namely, it is possible to recover optimal accuracy values when changing the UNets' architecture to increase their receptive field and match the image resolution (see Sup. Note 1, Table 2 and Fig. 6). Notwithstanding these updates, which require access to the code and programming skills, result in networks with a larger number of parameters, compromising computational requirements.*

Name	Kernel size	Stride	Receptive field	Trainable parameters	Equivalent downsampling
2D U-Net	3x3	1	48x48 pixels	1863375 (7.11MB)	1
2D U-Net D5K3	3x3	5	208x208 pixels	1863375 (7.11MB)	2^2
2D U-Net D2K6	6x6	2	211x211 pixels	6957519 (26.54 MB)	2^2

Table 1: *The network architecture and the training parameters for each dataset were preserved. The patch size for the input during training was dynamically changed with the image resizing to maintain the same field of view, except for the upsampling due to limited GPU capabilities. The values $2^{\{-2, -1\}}$ correspond to image upsamplings, 2^0 is the original image resolution and $2^{\{1, ..., 4\}}$ correspond to image downsampling.*

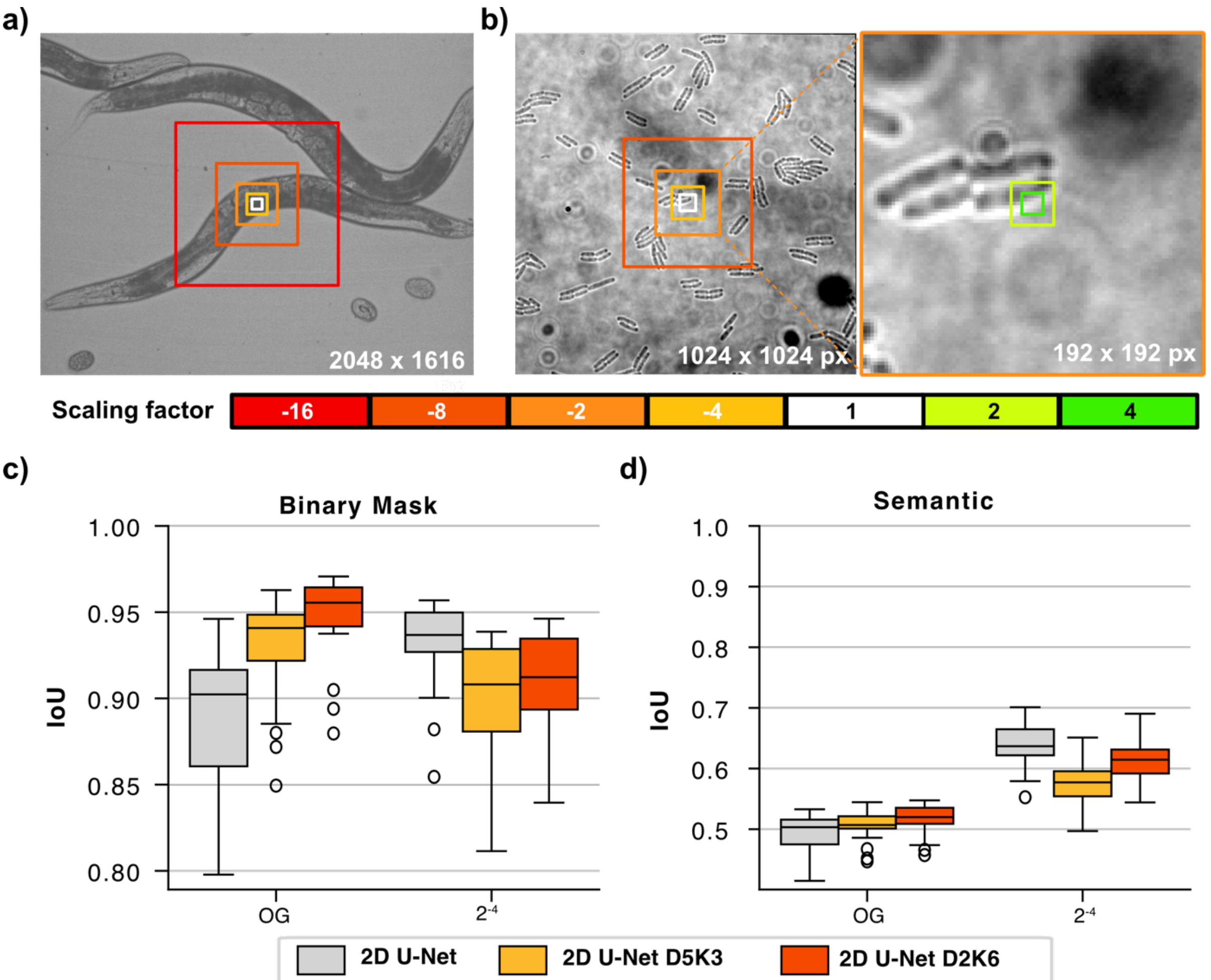

***Fig. 6**: **Receptive field with respect to the scaling factors**. A 2D U-Net with two pooling layers and a receptive field of 48 x 48 pixels was used to process a) C. elegans and b) E. coli datasets. When rescaling the images, the*

relationship between the receptive field and the image changes, being larger when downsampling the images and smaller when upsampling. c-d) U-Net architectures with two pooling layers and varying receptive fields were trained to segment C. elegans dataset at the original resolution and downsampled by a factor of 2^4 (a). Starting from the baseline U-Net with two pooling operations, we increased the effective receptive field by modifying only the convolutional kernel size and the dilation/stride parameters, thereby approximating the receptive field obtained when downsampling the input images (Table 1). Increasing the receptive field can recover segmentation performance comparable to image downsampling.

Our work intentionally focuses on a complementary practical perspective. Modifying the receptive field requires substantial deep learning expertise and architectural engineering, limiting reproducibility and accessibility for non-expert users. Indeed, as discussed in the manuscript, transformer-based architectures partially alleviate the receptive field limitations through improved long-range contextual modelling. However, our analyses (Fig. 3 i-j)) indicate that their performance also remains dependent on image resolution. In contrast, ReScale4DL was specifically conceived to support practical biomedical image analysis workflows, particularly within user-friendly deep learning frameworks where the network architecture and receptive field are often fixed and cannot be easily modified. We agree with the reviewer that in some cases, researchers cannot reduce resolution in their experiment; this is when downsampling provides an easy and user-friendly solution. For these reasons, we consider image rescaling to be a simple, architecture-agnostic, and broadly accessible strategy that complements, rather than replaces, more advanced receptive-field engineering approaches.

R2.2: When high resolution is required (for the object texture for instance), one could still downsample the image to perform segmentation. But then, would it be as easy as upsampling the masks to have a proper high-resolution segmentation. Examining Fig4, I would suspect that results could be underwhelming if downsampling was too important. It could be interesting to test until what resizing such an upsampling would be valuable.

Our response: We agree that when high resolution must be preserved for downstream measurements, segmenting a downsampled copy and upsampling the resulting mask is a sensible strategy. The new U2OS cell nuclei dataset illustrates this directly: segmentation accuracy at 60x is lower than at lower magnifications, and downsampling the 60x images significantly improves the result. The reviewer also asks how far this can be pushed. Our data suggest the practical limit lies around x4 upsampling: for both U2OS and S. aureus, accuracy already drops slightly at x4 upsampling, so x8 is expected to degrade further. For S. aureus this limit is also imposed by the optics, since the original images were acquired with a 63x objective and upsampling beyond x4 corresponds to a regime rarely encountered in conventional light microscopy.

R2.3: Going back to Fig 4, maybe I'm not understanding the plots c) but the text states "Only at coverage ratios exceeding 11% (downsampling factor > 2^3) did diameter estimates begin to diverge" while I'm feeling like the difference between the GT and prediction is smaller starting from 11%. Why? Fig 4 is also used to demonstrate that phenotypic discrimination is preserved even when downsampling occurs. The size difference between WT and Treatment is quite important (35 pixels versus 50 pixels) hence why the difference is still discriminable. I think the authors should add more conditions with varying radii (by changing the treatment concentration?) to identify the point at which differences become indistinguishable.

Our response: We apologise for the confusion in Fig 4c). After inspection of the results, we realised that the output of StarDist tends to include the periphery of bacteria while the manual annotations were slightly thinner. That is why the diameter distribution differs slightly between the ground truth and the predictions. This effect disappears at very low resolutions due to the low number of pixels available to

represent a cell. We included an example illustration in the supplementary material and changed the text accordingly to avoid these confusions as Fig. 7 (Supp Note 3).

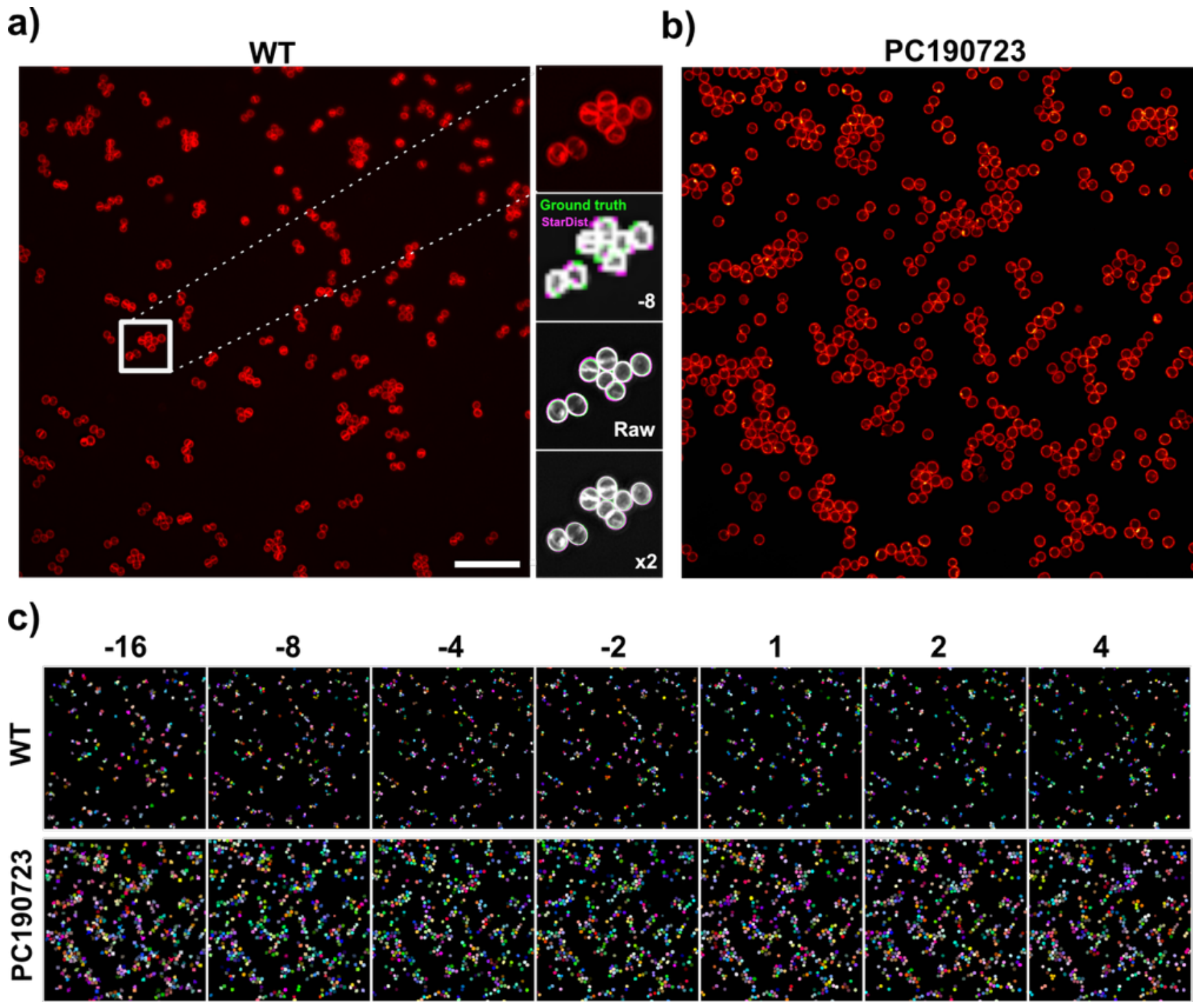

Fig. 8. Visual examples of S. Aureus segmentation results.) Original image of wild type (WT) S. Aureus and corresponding StarDist segmentation results (magenta) and ground truth (green) at different scaling factors. At higher resolutions (upsampling by a factor of 2), the StarDist model segments bacterial cells over a wider area than the manually labelled one. This difference decreases with lower resolutions (downsampling by a factor of 8). b) Original image of treated S. aureus. Scale bar 10µm in a) and b). c) Segmentation results for the FOVs in a) and b) at scaling factors ranging from −16 until 4.

We agree with the reviewer that it is interesting to identify which are the distinguishable differences depending on the resolution chosen, which is related to the Nyquist sample principle. If researchers aim to distinguish differences that are not representable with the chosen resolution (pixel sampling) or become indistinguishable, the imaging approach should most probably change. Also, these differences are commonly meant to be biologically relevant. In the case of S aureus, the difference between 35 and 50 pixels, which is almost doubling the size is related to the inhibition of division: bacteria cells double the size as if they will go into division, but they do not split. In this case, smaller differences, would not be physiologically relevant. Considering also the comment R3.1, we included some highlights in the discussion addressing these concerns:

> *"The proposed image rescaling with ReScale4DL is a simple, architecture-agnostic, broadly accessible strategy that complements, rather than replaces, more advanced receptive-field engineering. Importantly, ReScale4DL proposes a way to optimise imaging conditions considering the accuracy of image segmentation, which can be an intermediate step of an image analysis pipeline. For example, it can precede a cell tracking step, for which acquiring images at lower resolution can also be beneficial due to increased acquisition speed and field of view. Likewise, segmentation is often an intermediate step for further quantification within an area and for which a higher-resolution image is more convenient, such as measuring single-cell protein concentration or organelle distribution. In these cases, an optimal image resolution for the segmentation task can be achieved post-acquisition by using the ReScale4DL rescaling schema. Yet, researchers should always consider the Nyquist sample principle when deciding*

on their minimal imaging resolution to ensure that the features to be measured are adequately sampled."

R2.4: In [1], the authors developed a tool to determine the recommend receptive field size with respect to images/masks datasets: https://github.com/vinloo/u-net-receptive-field-study/tree/master/recommender-tool How does it relate to your % of coverage factor? What about resizing the pair of images/masks and running the tool. Does it give similar results as ReScale4DL? If yes, it would prevent training the networks.

Our response: We thank the reviewer for suggesting this valuable resource. We tried using the tool and with the code as it is, the environment is not reproducible nor the code worked well with our images. Yet, we modified slightly the recommender tool and run it on top of *S. aureus* dataset, which gave us the following output:

```
Current TRF size: 204
Mean RoI size: 43
Mean contrast level: 0.10
Contrast segmentable: True
------------------------------
Recommended TRF size: 54
```

As far as we understand, the TRF is an estimation of an optimal receptive field. In this case 54, is very similar to the 48x48 receptive field we have for StarDist and for which the original resolution was optimal, so we can say that both approaches lead to equivalent conclusions. Yet, we would like to highlight that in this case, it would prevent retraining the networks if and only if a user knows the size of their receptive field, which in the case of non-expert users may not happen.

R2.5: Finally, I think it's important that authors test their methods with images exhibiting lower SNR, as I'm suspecting that it could impact the downsampling factor.

Our reply: We agree that SNR can influence segmentation outcomes. The new U2OS cell nuclei experiments address this in a realistic setting, since acquiring the same field of view at 10x, 20x, 40x and 60x produces a genuine variation in SNR rather than a simulated one. Across these acquisitions, we again recover an optimal magnification that is consistent with the pixel-to-diameter ratio identified in the rest of the manuscript. External evidence points the same way: in Loos et al. (arXiv 2024) [1], the relationship between the target receptive field (TRF) and accuracy follows the same shape across high- and low-contrast datasets (Fig. 6a, e), with TRFs above 298 consistently yielding the highest accuracies (Table 2). The fact that this holds for both the high-contrast Lungs dataset and the low-contrast Nerve dataset supports our argument that image resolution exerts a stronger effect on network accuracy than SNR alone.

It is also worth clarifying the scope of the present study. ReScale4DL targets the imaging configuration, which is typically the limiting and expensive factor in an experimental pipeline. Within a given imaging modality, the dominant sources of SNR variance are usually staining and sample preparation rather than the acquisition settings themselves. A systematic evaluation of how different fluorophores, illumination sources or excitation regimes affect the optimal scaling factor is a worthwhile follow-up, but lies outside the scope of this work.

R2.6: In Fig3-4, it could be interesting to add the receptive field size and the image size (in addition to the % diameter per pixel).

Our reply: We included a figure (see the answer to R2.1).

R2.7: In Fig 4, I would like to see images for all the conditions, in order to see the segmentation results of the 37% and 44%.

Our reply: We included examples of these in the Suppl. Note 3, Fig. 7 (also copied in the answer to the comment R2.3).

R2.8: Why do you use diameter as a quantifier? This seems suboptimal for bacteria that are elongated. In addition, you have access to the masks, so using the area seems more straightforward and accurate.

Our reply: We intentionally used the diameter as it better captures the minimum spatial feature size that the network must resolve to distinguish forward from background regions. On the other hand, successful segmentation depends on the ability of the network to identify transitions between foreground and background within a limited contextual window. In this sense, the object diameter represents a highly restrictive dimension governing segmentation difficulty, and it can directly relate the image resolution and contextual information. For example, knowing the object diameter it can already inform about the minimum resolution needed to represent an individual object. Likewise, it can be informative for a good estimation of the receptive field. This can be observed in our datasets: although C. elegans worms occupy substantially larger image areas than E. coli, the optimal segmentation performance in both cases is obtained at comparable effective diameter ranges in pixels.

Reviewer #3 (Remarks to the Author):

In this paper, the authors report the effect of rescaling image data on the accuracy of deep-learning-based segmentation. Quantitative comparison of the accuracy clearly shows that there is an optimal level of image rescaling that improves the accuracy. Preprocessing by rescaling image data has been known as a trick among image analysts, but this paper presents concrete evidence of the advantage of this preprocessing step. Overall, the paper meets the general interest of the biological community where bioimage analysis using deep learning based methods is becoming standard, especially since it affects the design of the upstream imaging workflow design as shown in Fig.2.
The paper is associated with sample image data, reproducible processing codes to systematically rescale image data, parameters required for deep-learning-based segmentations, and reproducible analysis codes for plotting the accuracies with different levels of rescaling. With modifications and corrections of code in the GitHub repository explained below, I recommend this paper to be published in Nature Communications.
Overall Comments

R3.1: The paper shows that rescaling the original image to a smaller image increases the accuracy of image segmentation using a CNN-based algorithm. This is an advantage for measuring morphological parameters of the segmented region. However, the delineated region by such a segmentation step is often used for measuring other features, such as intensities and object distribution within that region in a different channel. It would be better to have some explanation of the applicability of the rescaling step in such workflows. Would it be better to avoid rescaling in such a multi-channel measurement case, or does the advantage of increased segmentation accuracy exceed the disadvantage of the loss of resolution in other channels?

Our reply: We thank the reviewer for highlighting this aspect, as it is an important consideration when planning the microscopy experiment. Besides the overall accuracy of the segmentation method, researchers would need to think about the entire pipeline (i.e., the use of the segmentation masks for further quantifications) and consider whether reducing the image resolution should be avoided and

instead, compute the downsampling after the acquisition. We have added a new paragraph in the discussion that reads as:

> *"The proposed image rescaling with ReScale4DL is a simple, architecture-agnostic, broadly accessible strategy that complements, rather than replaces, more advanced receptive-field engineering. Importantly, ReScale4DL proposes a way to optimise imaging conditions considering the accuracy of image segmentation, which can be an intermediate step of an image analysis pipeline. For example, it can precede a cell tracking step, for which acquiring images at lower resolution can also be beneficial due to increased acquisition speed and field of view. Likewise, segmentation is often an intermediate step for further quantification within an area and for which a higher-resolution image is more convenient, such as measuring single-cell protein concentration or organelle distribution. In these cases, an optimal image resolution for the segmentation task can be achieved post-acquisition by using the ReScale4DL rescaling schema. Yet, researchers should always consider the Nyquist sample principle when deciding on their minimal imaging resolution to ensure that the features to be measured are adequately sampled."*

R3.2: Another idea, instead of rescaling, is to adjust the size of the reception field in the CNN. This might be naturally avoided because the effect is too much, but it can be discussed why this was not an option.

Our reply: We totally agree with the reviewer and have addressed this comment in the answer to reviewer 2 (R2.1).

R3.3: Throughout the paper, a measure called "% diameter per pixel" is used to show the extent of rescaling. As the quality of the segmentation results relies on the relative size of objects, this may be a good indicator to gauge the expected accuracy of segmentation results. However, the use of this measure in the results was confusing to me. It would be better if the rescaling value were directly shown instead of % diameter per pixel, because these percentage values change depending on the size of the segmentation target, even when the rescaling value is identical. Especially when two different populations with differences in diameter are compared, such as the case in Fig. 4, this confusion is maximized. In Fig. 4a, wild type 22.4% diameter per pixel and treated 19% diameter per pixel probably are with the same rescaling level, but as the corresponding % diameter per pixel values become different, it was confusing and made me wonder why these two seemingly different conditions can be compared. In Fig. 4c and d, the horizontal axis is % diameter per pixel for showing the difference between the two populations; only the % diameter per pixel of one of the populations is shown in the axis label. In the GitHub Jupyter notebook Evaluate_Segmentation.ipynb, the other population is shown - I think the authors are slightly confused as well. To overcome such confusions, it is better to directly show the rescaling values (2, 2^2, 2^3 ...) in these figures, and add a new table to list these rescaling values and % diameter per pixel to show their relationship. For example:
Rescaling | % pixel diameter WT | % pixel diameter Treatment
2^0 | 2.8 | 2.4
2^1 | 5.7 | 4.7
2^2 | 11.4 | 9.5
2^3 | 22.9 | 19.0

Our response: We followed the reviewer advise and included new tables with the relationship between the image rescaling and the percentage of an object diameter covered by a pixel. This information is available in the new Figure 3 (see the answer to R1.3) and the Table 3 in the Supplementary Note 3, which looks as follow:

S. aureus	Pixel Diameter [%]	
**Rescaling factor**	**Wildtype**	**Treatment**
2^2	0.71	0.5
2	1.41	1.0
1	2.84	2.1
2^-1	5,67	4.1
2^-2	11,35	8.2
2^-3	22,73	16.5
2^-4	45,20	32.8

Table 3. Rescaling factors for S. Aureus. *The rescaling factor indicates data upsampling or downsampling, depending on whether it is higher or lower than one,respectively. Pixel diameter [%] refers to the proportion of the diameter covered by one pixel for the wild type and treated bacteria samples.*

Minor Comments

R3.4: page 1 Fig.1c: In this figure, there are three circular gray icons: 1) with two small white circles, 2) flat gray, and 3) with a broad black rim. There is no explanation about these icons, but it seems that these icons each represent 1) "fragmented segmentations caused by non-continuous edges and inner pixels misclassified as background, referred to as false negatives", 2) almost perfect segmentation, and 3) "over-generalization at edges". I think these icons and these explanations are better to be explicitly linked, as the same icons are also used in Figs 3a and c.

Our reply: Figure 1's caption now has the following additional text:

> *The icons represent in order from left to right: 1) fragmented segmentations caused by non-continuous edges and inner pixels misclassified as background (i.e., false negatives), 2) accurate segmentation, and 3) over-generalization at edges.*

R3.5: page 3 Fig 3b, d: What do the white circles in the plots represent?

Our reply: White circles are outliers that lie below the quartiles. We have clarified it in the figure caption (see the updated version of the figure and its caption in the answer to comment R1.3).

R3.6: page 3 Fig 3 legend "binary mask (merged label)": It took me some while to figure out that this means "merged = edge + inner region". Maybe add a bit more like: "binary mask (merged label = edge + inner region)"?

Our reply: This is now clarified in the legend as well (see the updated version of the figure and its caption in the answer to comment R1.3)

R3.7: Page 4 column 1, 2nd paragraph: missing Note number and Figure number

Our reply: We have reviewed the cross-references to supplementary notes and figures, and hopefully this is now solved.

R3.8: Page 4 column 2, "segmentation networks" and "Evaluation": missing Table number

Our reply: Same as in R3.7.

R3.9: Page 4 column 2, "the diameter Dij of non-rounded shapes (C. elegans and E. coli datasets) was computed as the median of the Euclidean Distance Transform (EDT) values contained in the object's skeleton. " It would be great to have the script used for this measurement in GitHub, and references, if existing, that use this seemingly new method for determining the diameter of elongated shapes.

Our reply: The code used for the analysis in this manuscript is publicly available in ReScale4DL GitHub repository. In particular, the calculation the reviewer asked for is available here: https://github.com/HenriquesLab/ReScale4DL/blob/main/src/rescale4dl/metrics/metrics.py#L1131

R3.10: There is a bug in the GitHub repo, Rescale_Images.ipynb. Line 124: all parameters should be capitalized to use the values defined in Line82-86. (https://github.com/HenriquesLab/ReScale4DL/blob/dc18418a61de9072bfb22ad18674195ff8e1cf8d/Notebooks/Rescale_Images.ipynb#L124)

Our reply: We thank the reviewer for identifying this error. It is now corrected.

R3.11: Supplementary Figure 5. The results shown in this figure are not associated with method details, and the explanation and interpretation of these results are immature. I could not understand how this was done or where I should focus. My impression is that this figure should be in a different new paper. Otherwise, it should be a part of the main text, not as a supplementary, as this figure itself seems to be a new finding, not appropriate for being "supplementary" to what is shown in the main text.

Our reply: We have integrated the results within the main text, within the first section of the results called "High resolution and segmentation accuracy: a counter-intuitive relationship". We agree with the reviewer that these results represent a novel finding and we consider them important examples of the impact that image resolution has overall, in NN-based approaches. By presenting together the segmentation results obtained using 2D, 3D, and foundation-model-based approaches, we believe the revised figure now provides a broader and more coherent overview of the impact of image resolution on segmentation performance across trainable neural network architectures.

REVIEWER COMMENTS

Reviewer #3 (Remarks to the Author):

I thank the authors for responding to my comments and reflecting my suggestions in the revised manuscript. The manuscript is ready for publication.

Aside from the manuscript itself, the code in GitHub does not seem to be working well. I added details of my experiences with three Jupyter notebooks listed in the README in below. The publication will be fully ready when these issues are solved. Asking a third person to test notebooks without giving any instructions might help fix these problems.

R3.1: 2. Evaluate_Segmentation2D.ipynb (The top README needs to be updated):

with INPUT_DIR = "Examples/segmentation_results/Saureus_instance_segmentation"

The 2nd cell returned following error

[errors reported by the reviewer]

3. Examples/data2rescale/Saureus_instance_segmentation/Images

The 3rd cell returned the following errors

[errors reported by the reviewer]

Our reply: We thank the reviewer for testing the notebooks and identifying the documentation problem. We corrected the README (https://github.com/HenriquesLab/ReScale4DL) links and added videos showing how to select the input folders and run the cells in order. We also tested the revised workflows from a clean installation using the bundled examples. The image-rescaling, 2D-segmentation, and foundation-model notebooks completed without errors and generated output images, plots, and summary statistics.
